# Development of transversal skills in higher education programs in conjunction with online learning: relationship between learning strategies, project-based pedagogical practices, e-learning platforms, and academic performance

**DOI:** 10.1016/j.heliyon.2024.e41099

**Published:** 2024-12-09

**Authors:** Yolanda Guerra-Macías, Sergio Tobón

**Affiliations:** a Universidad Autónoma de la Ciudad de México (UACM), Mexico; b Centro Universitario CIFE, Cuernavaca, Mexico

**Keywords:** Generic competencies, 21st-century skills, Virtual education, Higher education, Socioformation, Generic skills, Socioformative rubrics

## Abstract

This study investigates the development of transversal skills and their association with academic performance in university students enrolled in on-campus programs with online activities. A cross-sectional, descriptive, and quantitative research was conducted with 252 students from a public university in Mexico. Transversal skills, socioformative project-based practices, learning strategies, and the relevance of online activities were assessed using validated rubrics. The results indicated a low level of development in three transversal skills: research, entrepreneurship, and English, with the latter being the poorest rated. Critical and creative thinking exhibited the highest level of development. In the didactic component, socioformative project-based pedagogical practices and learning strategies showed acceptable levels. Students expressed satisfaction with complementary online activities, showing a preference for interactive videos and short videos under 4 min. Regression analysis and structural equations were used to examine the relationships between various factors. Results demonstrated that socioformative project-based pedagogical practices, learning strategies, and online education positively correlated with the development of transversal skills. Furthermore, a higher level of transversal skills was associated with better academic averages among students. Socioformative project-based pedagogical practices also correlated with academic performance through transversal skills. The study concludes that integrating online activities into on-campus programs, based on the socioformative pedagogical model, can enhance the development of transversal skills and improve academic performance. Further research into the implementation of this educational model and its long-term impact on university education and professional success is recommended.

## Highlights

• Research, entrepreneurship, and English proficiency levels are low.
• There have been advancements in socioformative practices such as inclusion and problem-solving.
• Students prefer interactive and short videos.
• Students are not interested in long videos or chat-based formats.
• Transversal skills predict university academic success.

## Graphical abstract

**Figure:**
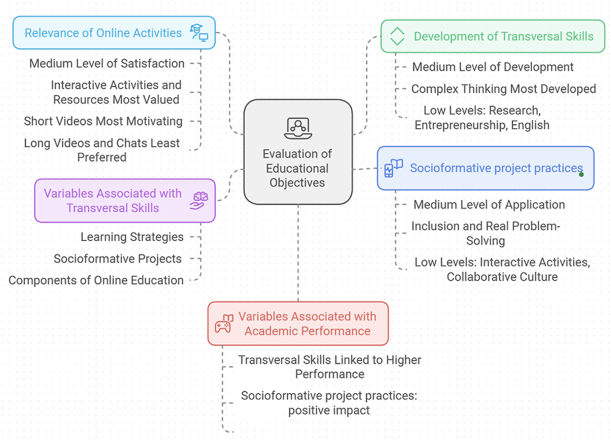

## 1 Introduction

For several decades, generic competencies (GC) have been a central axis in higher education policies, and many universities have incorporated them into graduate profiles and curricula [[1], [2], [3]]. Research in this area is increasing [4]. These types of competencies are closely related to current demands in professional practice, which focus on more general and less specialized training, with greater flexibility to adapt to the new environments and tasks required by the knowledge society. Therefore, generic competencies are considered important for professional and work success, in addition to specific competencies [5]. However, the concept of generic competencies is problematic because it tends to be addressed in different ways, without a common framework [6] and with much fragmentation in its approach. The latter is observed in the high number of capabilities associated with them, as presented in the Tuning Project [7].

In light of these issues, we propose reconceptualizing generic competencies as integral transversal skills within the pedagogical model of socioformation. In this way, they can be redefined as practices aimed at solving contextual problems in multiple areas and fields, articulating various capabilities, knowledge, and values, with ethics and community commitment [8]. Consequently, these are contextualized actions that are addressed systemically, without fragmenting their components, and that respond to the challenge of comprehensive training, considering social, economic, political, technological, and scientific challenges, among others, such as the emergence of artificial intelligence. To this end, they are articulated with the training processes of students [9,10] and teachers.

Several studies have analyzed the development of generic competencies in university students, but these studies have been in 100 % face-to-face or 100 % virtual programs [[11], [12], [13]]. There is very little information on the development of these competencies in face-to-face programs that are supported by virtual activities through a Learning Management System (10.13039/501100000608LMS). In this area, studies have generally focused on some aspect, such as the relationship with other variables, differences based on gender and field of study [14], and academic performance. Analyses that consider the relationship between different variables in a unified manner are lacking. Furthermore, although the impact of socioformation in Latin American higher education is increasingly being studied, there is little research on its relationship with transversal skills. This could provide guidance for the growing number of universities that are gradually developing flexible face-to-face university programs, integrating a virtual component, as an evolution to the changes generated in emergency remote education that they applied at the beginning of the COVID-19 pandemic.

Therefore, the present research had the following objectives.

1. To evaluate the level of development of transversal skills in students of face-to-face university programs who participate in online activities, identifying those skills that present higher and lower levels of development.
2. To analyze the implementation of teaching based on socioformative projects in face-to-face university programs with online activities, determining the relevance of these practices from the students' perspective.
3. To examine the relevance of online activities (through an LMS platform) as a complement to the training provided in face-to-face undergraduate university programs, evaluating their organization, ease of use, and perceived impact on student learning.
4. To identify and analyze the variables associated with the development of transversal skills in students of face-to-face university programs with online activities, determining which of these variables have a greater impact.
5. To determine the variables associated with the academic performance of students in face-to-face university programs with virtual activities, evaluating how these variables influence academic performance, success in study, class attendance, and student retention.

## 2 Literature review

### 2.1 Socioformation: an alternative to constructivism and connectivism

An approach that has influenced the educational models of many universities is constructivism, which at certain times has been predominant and still has a great influence. In this model, the educational work is focused on the student and the learning process, not on teaching, and for this, it works based on exploration, inquiry, and discovery with the continuous guidance and mediation of the teacher. In a more recent version, called social constructivism, the social, historical, environmental, and economic context is integrated into learning, along with peer interactions, to achieve more relevant learning. However, in this latest version, the axis of the process remains the individual student, even if approached from a social perspective. Constructivism and social constructivism present several problems and gaps: 1) both approaches focus on individual learning rather than transforming community problems; 2) while social constructivism addresses collaboration, it's primarily to achieve relevant learning for each student, not as a process in itself. This approach fails to consider learning at the team, organizational, or community level; 3) the goal is centered on achieving learning itself, rather than broader social development.

A more recent pedagogical model that is beginning to be applied in universities is connectivism, which is presented as an alternative to overcome the gaps in constructivism and social constructivism. Its central thesis is that learning occurs through connections between people, teams, and technology. It posits that not only individuals learn, as constructivism and social constructivism proposed, but also teams, groups, organizations, and technology itself. This has led to its adoption by many universities in the face of the emergence of the network society and digital technology. Some gaps in connectivism are: 1) it focuses on learning, even if it is group and machine learning, not on training or on transforming communities towards sustainability, coexistence, and inclusion; 2) it does not have an approach to transform the curriculum towards an inter- and transdisciplinary approach; and 3) there is little development of didactic strategies that have a connectivist nature.

Based on the gaps in constructivism and connectivism described in the previous paragraph, socioformation is proposed, a model proposed by Tobon [15] to guide new educational models in universities. This new training proposal emerged in Latin America based on the collaborative work of teachers from different educational levels, and has as a shared vision to contribute to overcoming the structural problems in the Region within the framework of sustainable social development, through work with the millennium goals in different organizations (community, business, and civil society). In summary, socioformation consists of training for social development with sustainability through problem-solving at the local level with a global vision, through collaborative culture and transversal projects [16]. This pedagogical model articulates digital technology as part of social development with sustainability, to promote quality of life and coexistence, not as an end in itself.

The characteristics of socioformation are as follows: 1) it focuses on collaborative culture, not on learning, competencies, connections, or cognitive processes; 2) it seeks to train based on solving problems that communities have, in order to develop sustainably, not based on academic tasks or activities in themselves; 3) it has as its center the transformation of organizations and communities, not the individual; 4) its main didactic strategy is transversal projects with inter- and transdisciplinarity, not learning based on organized and hierarchical topics; 5) it aims for the teacher to be a community or organizational mediator, not a facilitator of academic content; 6) it addresses ethics as a common element to all activities; and 7) evaluation is collaborative, aimed at solving problems considering the objectives of sustainable development.

### 2.2 From learning to socioformation

The traditional concept of learning has proven inadequate in addressing the complex challenges faced by individuals and society today. This notion, rooted in outdated educational paradigms, tends to reduce the formative process to mere acquisition of knowledge and skills, without considering the ethical dimension and social impact of this process. Learning, as conceived until now, presents significant gaps by failing to contemplate the comprehensive formation of the individual and their capacity to transform their environment. Moreover, its predominantly individualistic approach ignores the collaborative and systemic nature of knowledge construction in the digital era. This reductionist view of learning fails to address the complexity of contemporary global challenges, such as environmental sustainability, social inequality, or rapid technological evolution. Consequently, the need to transcend this limited concept and seek new paradigms that respond more effectively to the demands of a constantly changing world and promote the holistic development of individuals in harmony with their social and environmental context becomes evident.

**Fig. 1:**
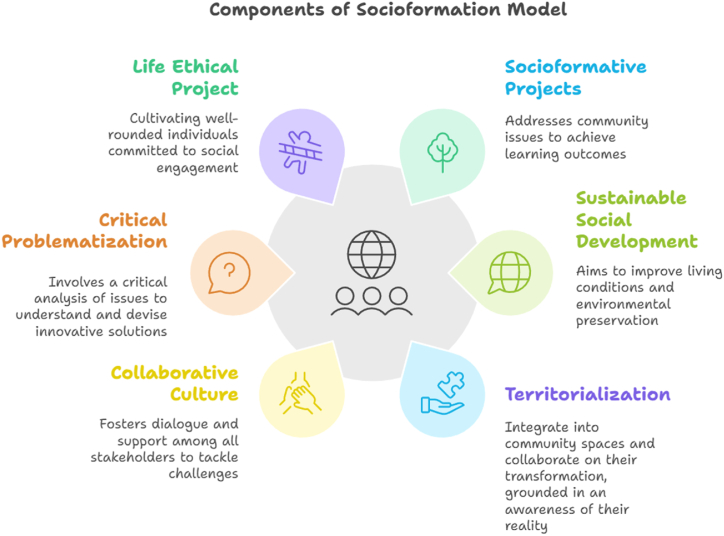
Essential axes of the socioformation pedagogical model.

Given the limitations of the learning concept, there emerges a proposal to transition towards a broader and deeper understanding of the formative process, embodied in the concept of socioformation proposed by Tobón [15]. This innovative approach recognizes formation as a comprehensive process that goes beyond mere knowledge acquisition, encompassing the development of all dimensions of the human being: cognitive, emotional, social, ethical, and praxeological. Socioformation is grounded in the construction and realization of an ethical life project, which not only seeks personal growth but also commits to social transformation and environmental preservation. This paradigm emphasizes the importance of forming individuals capable of facing the challenges of the knowledge society with creativity, critical thinking, and a strong sense of social responsibility. Socioformation promotes collaborative learning, dialogue of knowledge, and resolution of real-context problems, preparing individuals to be agents of change in their communities. By adopting this approach, it seeks to overcome the fragmentation of knowledge and foster a systemic vision that allows addressing the complexity of contemporary social and environmental phenomena, thus contributing to the construction of a more just, equitable, and sustainable society (Fig. 1).

### 2.3 Community and territorialization

The traditional concept of community has been subject to criticism for its limited and superficial approach. Historically, it has been conceived as a simple group of people sharing a geographical space, without considering the depth of relationships and purposes that should unite them. This reductionist view ignores the importance of dialogue, social cohesion, and collective action. In response to these limitations, socioformation proposes a renewed and more comprehensive concept of community. This new perspective understands community as a group of people who not only share a territory but also unite around common purposes, recognizing and accepting individual differences. The word "community" derives from "common" and "unity," reflecting the idea of a union based on shared elements. In this approach, community is characterized by inclusion and continuous dialogue as tools to resolve conflicts and problems, fostering collective empowerment to identify and transform adverse situations. It is crucial to understand that community encompasses all social actors: students, teachers, authorities, educational institutions, families, the general population, organizations, and the environment itself. This holistic vision recognizes the interconnection of all these elements and their fundamental role in building a resilient and transformative community, capable of facing contemporary challenges collaboratively and effectively.

It is necessary to address the community in its territory. The concept of territory in socioformation goes beyond a simple geographical delimitation, encompassing the complex relationships between people and their physical and social environment. Territorialization, as a formative process, implies a deep immersion in the space where the community lives, seeking an empathetic understanding of its reality and the problems it faces in daily life. For example, when addressing drug addiction in a community, territorialization would lead us to examine not only the problem itself but also its relationship with the family economy, the formation of criminal groups, and power structures in micro-trafficking. This approach seeks to understand the problem in its diversity of relationships, foster awareness, and generate empowerment for social transformation. In contrast, contextualization, although valuable, is limited to relating learning content to potentially significant situations in the environment, without necessarily promoting critical analysis or transformation of living conditions. While territorialization involves a deep analysis of the relationships between people and their environment to detect complex problems, contextualization is based on situated learning from the learners' prior knowledge. For instance, contextualization might simply relate learning outcomes to familiar topics such as families or local businesses, without necessarily addressing underlying issues or promoting significant social change.

### 2.4 Problems with generic competencies

Pedagogical models in universities have been built in the last two decades under the influence of the competency approach, as a consequence of the path traced by the World Conference on Higher Education in 1998 [17], which suggested following this path to improve the quality of education, and this promoted the establishment of public policies in Latin America aimed at incorporating competency-based training in the curriculum explicitly or implicitly. This has led universities to incorporate this approach massively, either as an institutional policy or an initiative of their teachers. In the case of Mexico, there are few universities in which competencies are not addressed in at least one department, and top-level universities have incorporated them into their study plans based on the initiative of their academics and authorities [[18], [19], [20]]. However, this approach has several problems. First, it is proposed from theory that they are a combination of attributes [19], but in practice they are addressed as separate knowledge or elements, and knowledge competencies, doing competencies, and value or attitude competencies are differentiated. Second, they seek to make learning more relevant [21], but they fall into an excess of formality and subdivisions that affect their implementation by teachers by increasing the administrative burden, due to the high number of subdivisions, such as: graduate profile competencies, subject competencies, competencies by topic, learning outcomes, criteria, indicators, etc. [22]. Third, they focus on individual learning [23] and do not address the learning of groups and communities. Although some authors have tried to give competencies a pedagogical approach, such as Díaz-Barriga [22], the problems are still present in this proposal.

Competencies are divided into two classes: specific (proper to a profession, trade, career, or field) and generic. The latter consist of abilities that people have to perform tasks and activities in various areas [24], based on knowledge acquired in different educational spaces. In the last two decades, they have been incorporated into international and national policies related to the improvement of higher education, as is the case of the Tuning project in Europe [25] Gonzales & Wagenaar, and Latin America [7,26], the 6x4 Project [27], the study of comparison of learning outcomes in higher education in different countries [28], or the Diagnostic Report on the Strategy of Competencies, Skills, and Abilities of Mexico [28].

However, in many cases generic competencies have remained only in discourse and it has been very difficult to address them with students, due to several gaps and problems in their approach and addressing. A first gap is that they are conceived, above all, as an end in themselves, and when they are not, they are justified to contribute to employability [5,29], mobility, or citizenship [26]. Very rarely are they conceived to contribute to the development of communities and organizations with sustainability, within the framework of inclusion, gender equity, social justice, and addressing diversity. Most commonly, in university curricula they are addressed in a decontextualized manner, without considering problem-solving to transform the social, economic, political, cultural, historical, scientific, technological, recreational, sports, or environmental environment, based on strategies that have an impact on the community, organizations, or companies, such as transversal projects. In some cases, what is proposed is to address them within the framework of subjects, based on the topics that are in these [30,31].

A second gap is that they tend to be addressed in a fragmented way, without a systemic thinking that integrates them, and that is why, generally, they are posed as skills or capabilities [1], without articulating other processes. This can be observed in the following examples of generic competencies [7]: "Ability to apply knowledge in practice", "Ability to organize and plan time", "Knowledge about the area of study and the profession", "Social responsibility and civic commitment", "Oral and written communication skills", among others. This same can be observed in the list of 21st century competencies, which is very broad and diverse, and addresses competencies such as listening skills, ability to communicate effectively, work respectfully and effectively, information literacy and media literacy, among many others [32]. It can be observed that they are not integrative processes of skills, knowledge, and values. This fragmentation leads to having a high number of generic competencies that makes their development and evaluation very difficult for teachers. For example, in the Tuning Latin America Project, 27 competencies were established [7], in the Tuning Project of the European Union, 30 competencies [26], and in a recent study, 40 generic competencies [33].

A third gap is that generic competencies tend to be addressed with multiple terms and concepts [34], such as employability skills, generic capabilities, key competencies, holistic competencies, basic competencies, soft skills, and transferable skills [1]. And every day new terms are proposed within this same area that increase confusion, such as meta-competencies [35], universal competences [36], and global competencies and trans-regional generic competences [33]. This diversity of concepts allows such different aspects as attitudes (positive attitude, empathetic attitude, motivation for quality work, sense of challenge, etc.), values (commitment, loyalty, responsibility, and honesty), skills (problem-solving, management, autonomy, creativity, etc.) and knowledge (knowledge of the environment, self-knowledge, and knowledge of others, etc.) to enter the same field, within generic competencies, with which the very concept of competencies ceases to apply, because various attributes would no longer be integrated to address tasks.

A fourth gap is that generic competencies often remain in general and abstract elements that are difficult for many teachers and students to understand and put into practice. Some examples of this type of description are: "Capacity for abstraction, analysis and synthesis" and "Interpersonal skills" [7,26] or "mastery of professional knowledge" and "methodology of the profession" [27]. This abstract language makes it difficult for university teachers who, in many cases, only have mastery of their profession and have little knowledge of pedagogy or the topic of skills, to address them. This makes it difficult to arrive at a coherent definition of generic competencies [37,38].

### 2.5 Rethinking generic competencies: transversal skills

Due to the problems described in generic competencies, socioformation proposes reconceptualizing the concept as transversal practices or transversal skills (TS), defined as integral and collaborative actions to solve problems in various contexts by articulating capabilities, knowledge, and values, with ethical values and commitment to the transformation of society [39,40]. As a shared vision, TS seek to contribute to the development of communities within the framework of sustainability [41], to have an impact on achieving the millennium goals, such as poverty reduction, health improvement, environmental care, prevention of global warming, employability [42], etc.

Some of the characteristics of TS are: 1) they do not focus on specific and punctual capabilities or skills, as tends to occur with generic competencies; 2) they are centered on problem-solving, not on addressing tasks or activities; 3) they are addressed in multiple areas and fields, not in a specific area [42]; 4) they are developed in various educational spaces and moments, not only in one subject or semester; 5) they imply that the curriculum changes from an organization by subjects to a flexible structure by collaborative projects; and 6) TS are established by articulating knowledge, while generic competencies are addressed for each knowledge (knowing, knowing how to do, knowing how to be, and knowing how to live together) and this affects their development in universities [15].

### 2.6 Face-to-face education with online activities as a complement or support in the training process

COVID-19 implied a profound change in higher education because universities had to move from face-to-face to distance teaching through different options and modalities. At the beginning, many higher education institutions had to implement emergency remote education, which consists of offering instruction to students without prior planning, in a crisis context and using basic or available resources in the environment [43], such as telephone, email, web pages, text messages, etc. This was due to students and teachers not having sufficient internet access [44], lacking e-learning platforms [45], and most teachers not having training to work online.

Due to this unexpected change in higher education, various problems began to arise for students in Mexico [45], such as: 1) increased dropout rates due to lack of technological resources, such as internet service and computers; 2) lower academic performance due to reduced and noisy spaces designated for study; 3) lack of good and adequate internet service; 4) infections of their family members or themselves; 5) having to work to cover family expenses; and 6) depression and discouragement due to lack of economic resources, health problems linked to the pandemic, or situations related to social inequality [46,47].

As the pandemic prolonged over time, universities began incorporating technological elements and gradually started implementing online education modalities [48,49], even with existing limitations [46], through progressive teacher training, the use of videoconferencing systems, the use of e-learning platforms, digitization of materials, and the use of existing educational resources on the internet and online platforms [50,51]. This is how some universities began to integrate online education activities within face-to-face programs, through the implementation of LMS platforms and videoconferences.

The COVID-19 era spurred digitalization and online education in many universities, and that remains a strategic objective in higher education [52]. However, knowledge about the development of transversal skills and their role in academic performance in face-to-face programs complemented with online training is scarce. Some research seems to indicate that during the pandemic, generic competencies weakened due to the problems faced by universities in having to adapt suddenly to online classes [53]. On the other hand, there is evidence of improvement in generic competencies from online courses (both intrapersonal and interpersonal) [54], but it remains to be determined the relationship with pedagogical practices assumed from socioformation and digital resources for learning (LMS platform and videoconferences).

### 2.7 Socioformative model of online education

The integration of socioformation in online education, complementary to in-person university programs, focuses on generating collaborative and inclusive learning communities oriented towards problematizing reality through critical thinking [8]. To this end, the virtual classroom is designed to be an easily accessible, comprehensible, and intuitive space that provides comfort to students and promotes the strengthening of their autonomy and self-regulation of learning. This process includes elements such as satisfaction with online activities, ease of use of the e-learning platform, and the availability of engaging activities and resources that motivate learners to log in and remain for the necessary time. The organization of the virtual classroom, through interactive and gamified activities, fosters adaptive and personalized learning. This model incorporates universal design for learning to offer diverse options that allow students to develop comprehensively and contribute to the transformation of their communities.

This model stands out for incorporating highly innovative aspects in higher education through online activities, with an emphasis on the hyper-personalization of learning and the advanced use of emerging technologies. From the socioformative perspective, highly flexible online education activities are prioritized, enhancing the development of transversal skills such as critical thinking, creativity, computational thinking, and the ethical life project, responding to the challenges of hybrid education, which requires constant adaptation and a student-centered approach. Furthermore, artificial intelligence plays a transformative role in personalizing the educational experience, optimizing evaluation processes, and offering immediate feedback, enriching the interaction between students and teachers. Tools such as immersive learning scenarios, based on virtual and augmented reality, not only reinforce student engagement but also improve knowledge retention and create meaningful educational experiences. These innovations prepare students for a professional environment based on digital culture, dynamic and constantly evolving, while driving educational institutions to redefine their pedagogical practices and teaching models.

### 2.8 Socioformative projects

Socioformative projects represent an innovative pedagogical strategy that seeks to transform the community environment through collaborative work between students and various social actors. Developed by Tobón [15], this methodology is based on problematizing the territory from multiple perspectives, promoting critical thinking and the articulation of knowledge to address real and urgent community problems. They are characterized by: 1) problematizing the territory from different perspectives to understand reality and identify the most urgent problems; 2) approaching from critical thinking, seeking to generate awareness about problems and the need for transformation; 3) promoting the articulation of knowledge based on critical analysis and knowledge management; 4) implementing through simple actions with minimal prior planning; 5) involving different actors from the educational institution and the community; 6) addressing digital technology as support to transform the community; and 7) emphasizing continuous formative evaluation based on transformation processes and collaborative improvement. Unlike other educational approaches, socioformative projects are characterized by emphasizing simple actions with minimal prior planning, thus fostering active and protagonistic participation of students in conjunction with community actors [8]. This methodology innovatively integrates digital technology as crucial support for social transformation, adeptly adapting to the various connectivity possibilities available in each context. A distinctive aspect of socioformative projects is their emphasis on continuous formative evaluation [16], where students assume the role of protagonists in the educational process through self-assessment and co-evaluation. This approach promotes the application of critical thinking to innovate and improve the community creatively, generating solutions adapted to local needs [13]. The flexibility and adaptability of this methodology make it particularly effective in addressing contemporary educational challenges in Latin America.

The methodology of socioformative projects is deeply rooted in Latin American culture, organically integrating fundamental aspects such as collectivism, rich cultural diversity, deep connection with the land, and the strong sense of familialism characteristic of the region [13]. Socioformative projects reflect and enhance the Latin American tendency to work in teams, uniting diverse strengths, talents, and resources to achieve a common purpose, as is typical in traditional communities of the region. One of the most prominent features of this methodology is its inherent flexibility in managing times and activities, harmoniously adapting to local cultural rhythms and placing special emphasis on interculturality as a transversal axis of the educational process [39]. This cultural orientation is palpably manifested in the methodological simplicity of the projects, in the implementation of flexible schedules that respect community dynamics, and in the marked emphasis on collective and collaborative work. These aspects resonate deeply with Latin American idiosyncrasy, facilitating a natural and effective appropriation of the projects by teachers, students, and community members. The socioformative methodology, being so intrinsically aligned with the cultural values and practices of Latin America, not only improves the effectiveness of the educational process but also strengthens the cultural identity and sense of belonging of the participants.

Socioformative projects differ significantly from other methodologies imported from Europe and the United States in several key aspects, reflecting their unique adaptation to the Latin American context. While Project-Based Learning (PBL) and the STEAM approach tend to focus on predefined academic objectives and measurable outcomes, socioformative projects prioritize comprehensive community transformation and local sustainable development (Table 1). Service-learning, although similar in its social orientation, does not necessarily integrate the cultural flexibility and deep sense of collectivism that are characteristic of Latin American socioformation. Problem-based learning, for its part, is often limited to simulated scenarios or case studies, in stark contrast to the direct approach to real and urgent problems that characterizes socioformation. The latter methodology stands out for its ability to adapt its processes to the specific rhythms and needs of each community, incorporating cultural elements such as familialism, personalism, and reciprocity, which are fundamental in the Latin American worldview. Moreover, while imported methodologies often require specific resources and rigid structures, socioformative projects are characterized by their flexibility and ability to be implemented with minimal resources, making the most of the social and cultural capital of communities. This adaptability and focus on social transformation make socioformation a particularly effective tool for addressing the unique educational and social challenges of Latin America.

**Table 1:** Comparison of socioformative projects with other project methodologies.

Aspect	Socioformative Projects	Project-Based Learning	STEAM	Service-Learning	Problem-Based Learning
Definition	Collaborative process to transform the community environment through solving real problems with critical thinking	Methodology that organizes learning around projects based on challenging questions or problems	Interdisciplinary approach integrating Science, Technology, Engineering, Arts, and Mathematics	Educational strategy that combines learning objectives with community service	Learning method in which students learn through critical analysis of problems
Emphasis	Social transformation and sustainable development of the community in its territory based on innovation	Acquisition of knowledge and skills through research and product creation	Integration of disciplines to solve real-world problems	Development of civic responsibility and strengthening of communities	Development of problem-solving skills and critical thinking
Origin	Mexico	United States (John Dewey, William Kilpatrick)	United States (Georgette Yakman)	United States (1960s–1970s)	Canada (McMaster University, 1960s)
Steps/Stages	1. Sensitization 2. Problematization 3. Transformation 4. Socialization	1. Driving question 2. Planning 3. Research 4. Product creation 5. Presentation 6. Evaluation	1. Problem identification 2. Research and design 3. Prototyping 4. Testing and refinement 5. Communication of results	1. Needs identification 2. Design and planning 3. Execution 4. Evaluation	1. Problem presentation 2. Information inquiry 3. Problem analysis 4. Proposal of options to address the problem 5. Reflection
Benefits	- Local sustainable development - Strengthening of cultural identity - Real problem-solving skills - Ethical and civic commitment	− 21st-century skills - Intrinsic motivation - Deep learning - Project management skills	- Integrated thinking - Creativity and innovation - Preparation for future careers - Technical and artistic skills	- Promotes social awareness - Development of empathy - Civic skills	- Critical thinking - Self-directed learning - Teamwork skills
Cultural characteristics considered	- Collectivism - Flexibility - Reciprocity - Connection with land and community - Empathy with the community	- Formalism - Results orientation - Innovation - Planning	- Multiculturalism - Global focus - Technological innovation	- Structured altruism - Social responsibility	- Rationalism - Critical questioning - Learning autonomy - Emphasis on cognitive aspects

### 2.9 Learning strategies: a socioformative perspective

Learning strategies, traditionally defined by authors such as Weinstein and Mayer [55], have been conceptualized as techniques and procedures that students employ to acquire, retain, and retrieve information, thus focusing on cognitive learning itself. From the socioformative perspective, however, learning strategies transcend this instrumental view to become systematic processes of collaboration, and cognitive and metacognitive mediation to help each person achieve comprehensive human formation, aiming for citizens to become sensitized and assist in the transformation of their community to improve living conditions. These processes are grounded in understanding how the brain learns and adapts, incorporating principles of neuroeducation to optimize the acquisition and application of knowledge. Socioformative learning strategies seek to develop complex thinking and the ability to solve real-context problems, considering principles of universal design for learning to ensure that all students can access and benefit from the formative process, regardless of their individual characteristics or preferred learning styles.

Socioformation innovates by reframing learning strategies as tools for social transformation and not just individual development, to contribute to forming citizens who can live and participate in the knowledge society and digital culture, in a world increasingly based on artificial intelligence. Unlike traditional approaches centered on information processing, this paradigm emphasizes collaboration, context problematization, and continuous self-assessment as key elements in forming citizens who contribute to the sustainable development of their communities. Learning strategies from the socioformative perspective, such as case analysis, collaboration, and the use of digital technology based on artificial intelligence, promote critical thinking and metacognition through real challenges, using emerging technologies and building collective knowledge that positively impacts their social environment. This approach innovatively integrates transversal skills, preparing students to navigate and contribute significantly in an increasingly technological and complex world, without losing sight of the importance of comprehensive human development and commitment to collective well-being.

## 3 Methodology

### 3.1 Type of study

A cross-sectional [56], descriptive, and quantitative study was conducted to diagnose the level of development of transversal skills from socioformation in university students of face-to-face programs that had online activities as a complement and support. To this end, we sought to identify the association of these transversal skills with academic performance and factors related to their development. Thus, descriptive analyses were made, comparisons between groups were established, and associations between variables were determined through the use of structural equations.

### 3.2 Participants

The study included 252 students from various undergraduate programs at a public university in Mexico City. These programs combined face-to-face instruction with online subjects and activities. They belonged to the areas of Humanities and Social Sciences, Sciences and Humanities, and Science and Technology. 63 % were women, and 37 % were men. The age ranged from 18 to 61 years, with a mean of 26.5 years and a statistical deviation of 6.6 years. Students located in the sixth semester of their careers predominated (median of 6). The sample was non-probabilistic for convenience, and students were contacted through email, based on a university database. The instruments were completed through a Google Forms form at the end of 2020, and permission was obtained by email to use them in the present investigation. All sample participants completed the instruments.

### 3.3 Instruments

In this study, four instruments previously designed by Tobón [57] were utilized. Following their application to 252 students, evidence of validity and reliability was gathered. Initially, a confirmatory factor analysis was conducted to determine if the factors aligned with theoretical expectations and to identify factor loadings [58], employing diagonally weighted least squares (DWLS) due to the ordinal nature of the instruments [58]. Subsequently, the goodness of fit for the four instruments was assessed, considering normalized chi-square values (χ2/df < 3), the Tucker-Lewis Index (TLI > .90), the Comparative Fit Index (CFI > .90), and the root mean square error of approximation (RMSEA < .08). All analyses were conducted using the R software, version 4.3, primarily utilizing the lavaan package. Finally, reliability was evaluated using Cronbach's alpha coefficient and the composite reliability for each instrument [59].

*Transversal Skills Rubric*. This is an instrument developed by Tobon [57] that evaluates 10 essential transversal skills in university students, which are described in. Its purpose is to determine the level of development of transversal skills assumed as practices, in secondary, middle, and higher education students. Some of these competencies are: digital technology, research, socio-emotional skills, among others. In the present study, through confirmatory factor analysis, it was found that the rubric has a single factor, consistent with what was expected at the theoretical level, and all items presented factor loadings higher than 0.5 [58]. The goodness of fit indices were within the expected ranges: X^2^/df = 2.472; GFI= 0.983; RMSEA = 0.022, RMR = 0.056; CFI = 0.995; NFI = 0.959, TLI = 0.994. The average variance extracted was 0.298, and both the reliability measured by Cronbach's Alpha (0.80) and the composite reliability (0.805) presented adequate levels.

*Teaching Based on Socioformative Projects Rubric*. This instrument, developed by Tobon [57], evaluates 10 types of practices through which socioformative projects are implemented, even if a university does not explicitly follow this methodology. The purpose is to determine the level of application of a series of explicit or implicit pedagogical tools in the training process guided by teachers, but valued by students to achieve the expected learning outcomes in the curriculum. Some of these practices related to socioformative projects include: community issues, collaborative culture, interactive and dynamic activities, formative assessment, inclusion, etc. (see Table 3). The rubric was found to possess two factors, consistent with theoretical expectations, and all items presented factor loadings greater than 0.5 [58]. The goodness-of-fit indices were within the expected ranges: χ^2^/df = 0.476; GFI = 0.997; RMSEA = 0; RMR = 0.034; CFI = 1; NFI = 0.993; TLI = 1. The average variance extracted was 0.63. Both the reliability measured by Cronbach's Alpha (0.905) and the composite reliability (0.94) presented adequate levels.

*Socioformative Rubric for Learning Strategies*. This is an instrument designed by Tobon [57] and evaluates 9 learning strategies in students from the socioformative pedagogical model. The purpose of the instrument is to determine the level of mastery and implementation of each strategy by students, based on a problem, challenge, or contextual situation. Some learning strategies that are evaluated are: class preparation, class participation, information search, problem solving, among others. The factor analysis identified a single factor, called "learning strategies," and this was consistent with what was expected at the instrument design level. The factor loadings of the items were higher than 0.5 [58], and the goodness of fit measures were within the expected range: X^2^/df = 0.665; GFI= 0.994; RMSEA = 0, RMR = 0.043; CFI = 1; NFI = 0.989 and TLI = 1. The average variance extracted was 0.5. Both the reliability measured by Cronbach's Alpha (0.878) and the composite reliability (0.89) presented adequate levels.

*Online Education Relevance Scale*. This is an instrument developed by Tobon [57]. It is composed of 7 questions that seek to determine the degree of relevance, importance, and innovation of online education in face-to-face university programs. Additionally, it allows establishing the degree of development of virtual classrooms and the power of attraction of virtual activities for students. In the present study, a single factor was found in correspondence with what was established in the instrument design, and all questions presented factor loadings higher than 0.5 [58]. The goodness of fit data were optimal: X^2^/df = 0.729; GFI= 0.996; RMSEA = 0, RMR = 0.038; CFI = 1; NFI = 0.989 and TLI = 1. The average variance extracted was 0.45. Both the reliability measured by Cronbach's Alpha (0.820) and the composite reliability (0.83) presented adequate levels.

*Sociodemographic Factors, Learning Resources, and Academic Performance Questionnaire*. This is an instrument developed by Tobon [57], which evaluates various sociodemographic and academic factors in students, such as gender, age, economic conditions, semester, study modality, most satisfactory learning resources, least satisfactory learning resources, academic average, grades in different subjects, dropout, among other aspects. Because it has open and closed questions, and its purpose is to collect general information, its validation was not necessary.

### 3.4 Data analysis procedure

In the present investigation, the following statistical analyses were performed according to the established purposes.

1. Estimation of data normality. This was done using the values of skewness and kurtosis, establishing normality when the values are in a range of ±2.0 [59,60]. All variables met this criterion, and therefore parametric tests were used in the analyses.
2. Estimation of variable levels. To determine the level of development of transversal skills, the degree of application of teaching based on socioformative projects, the use of learning strategies, and the level of relevance of online activities in face-to-face university programs from the students' perception, the mean and standard deviation (SD) were determined [61,62]. Then, we sought to determine if the mean of each of these constructs and variables was equal to or significantly different from an expected theoretical value using Student's t-test for one sample [63]. For each variable, the expected theoretical value was 3.0, as it represents the average or acceptable level, based on the applied instruments [64,65]. Likewise, distribution ranges (μ ± 1σ) were determined to identify atypical values in the indicated variables (transversal skills, teaching based on socioformative projects, learning strategies, and relevance of online activities). For this, the average of the obtained means was taken as a reference, and according to the variation given by the standard deviation (σ), a normal behavior zone was established at 68 % [[66], [67], [68]].
3. Regression analysis. A multiple linear regression analysis was performed to identify those variables that predict transversal skills, considering the total measure and each competency separately. For this, the stepwise method was used, which allows determining the model and variables with greater association power, considering the value of the adjusted betas and a significance less than 0.05 [61,69]. Using this same method, we sought to identify if the variables of transversal skills, teaching based on socioformative projects, learning strategies, and relevance of online activities were predictors of academic performance variables, such as grades obtained in the semester, the number of subjects approved with high grades, the number of subjects not approved in the semester, and the number of times studies have been suspended, among other aspects.
4. Structural equations. We sought to determine if transversal skills and teaching based on socioformative projects have effects on academic performance variables such as grades for the current semester and the previous semester, the number of subjects approved with high grades, the number of subjects not certified in the previous semester, and semester suspension. These relationships were analyzed through structural equation modeling. In this regard, we sought to graphically describe the causal relationships between variables [70,71], for which AMOS v.23 software and the Unweighted Least Squares (ULS) estimation method were used [72]. The quality of the model was evaluated using the set of goodness of fit statistics proposed in the literature [73].

For all effects, a p-value <0.05 was considered statistically significant [74,75].

### 3.5 Ethical aspects

In the present investigation, the following ethical aspects were met: 1) participants were informed of the purposes of the study and were asked for written authorization before answering the questions on the online form (out of 256 respondents, four students did not agree to participate in the research); 2) the study was approved by the CIFE Ethics Committee in 2022, with the code (2021-012); 3) participants could withdraw at any time from the study without any pressure or question; and 4) the confidentiality of information was assured to participants based on the Mexican Law on Protection of Personal Data [76].

## 4 Results

### 4.1 Development level of transversal skills in on-site university programs with integrated online activities

Table 2 shows that the students had a level of development of the transversal skills significantly higher than 3.0 (expected mean level at the theoretical level) in most of the competencies evaluated, with the exception of English (2.39), Entrepreneurship (2.94) and Research (2.99) which presented a level significantly lower than the expected theoretical mean of 3.0. Regarding the extreme values, measured with the formula of the mean plus or minus one standard deviation (μ ± 1σ), Complex Thinking obtained the highest value (3.56) and English competency, the lowest value (2.39) Table 2.

**Table 2:** Level of development of transversal skills in students (n=252).

Transversal skills	Average (+DS)	Asymmetry	Kurtosis	*t*-test (Theoretical mean: 3.0)
1. Digital technology	3.01 (±0.755)	0.373	0.699	*t*: 63.353; *p < 0*.001
2. Social-emotional skills	3.40 (±1.116)	−0.165	−0.813	*t*: 48.381; *p < 0*.001
3. Collaborative work	3.35 (±1.146)	−0.276	−0.669	*t*: 46.454; *p < 0*.001
4. Management of continuous training	3.23 (±1.019)	−0.520	0.069	*t*: 50.319; *p < 0*.001
5. Complex thinking	**3.56∗**(+0.818)	−0.303	−0.207	*t*: 69.046; *p < 0*.001
6. Leadership	3.19 (±1.041)	−0.092	−0.092	*t*: 48.767; *p < 0*.001
7. Research	**2.99** (±0.791)	−0.188	1.100	*t*: 60.137; *p < 0*.001
8. Oral and written communication	3.38 (±0.965)	−0.606	0.409	*t*: 55.730; *p < 0*.001
9. Entrepreneurship	**2.94** (±1.002)	0.168	0.538	*t*: 46.576; *p < 0*.001
10. English	**2.39∗** (±1.041)	0.224	0.363	*t*: 36.482; *p < 0*.001
General rubric for transversal skills	3.144 (±1.052)	0.005	−0.298	*t*: 49.668; *p < 0*.001

Note: Sig. *t*-test, p < 0.05; distribution ranges: μ + 1σ = 3.459 and μ - 1σ = 2.828.

The values in bold were the highest and lowest in each of the transversal skills addressed.

Values with ∗ were very high and very low.

### 4.2 Implementation of teaching based on socioformative projects in face-to-face university programs with online activities

Regarding the teaching based on socioformative projects applied in on-site university programs with online activities, Table 3 presents the diagnosis of these practices, based on the Rubric used in the self-evaluation by the students. Most of the pedagogical practices presented a mean with a value significantly higher than the theoretical mean of 3.0 (medium or acceptable level). There were two pedagogical practices with values significantly below the minimum acceptable level (theoretical mean of 3.0), which were collaborative culture and interactive and dynamic activities. There were two pedagogical practices with a higher value than the others, which were inclusion (3.47) and **Community Issues** (3.45), which were identified from the formula of the mean plus or minus one standard deviation).

**Table 3:** Level of implementation of teaching based on socioformative projects of teachers in face-to-face university programs with online activities, evaluated by university students.

Teaching based on socioformative projects	Mean (±SD)	Asymmetry	Kurtosis	*t*-test (Theoretical mean: 3.0)
**1. Community Issues**	**3.45∗**(+0.15)	−0.345	−0.315	*t*: 55.088; *p < 0*.001
**2. Interactive and Dynamic Activities**	**2.92** (±0.97)	0.223	−0.535	*t*: 47.872; *p < 0*.001
**3. Inclusion**	**3.47∗**(+0.98)	0.003	−0.679	*t*: 56.333; *p < 0*.001
4. Critical analysis	3.21 (±1.17)	−0.284	−0.731	*t*: 43.451; *p < 0*.001
5. Student-Directed Class Management	3.07 (±1.21)	−0.084	−0.840	*t*: 40.228; *p < 0*.001
**6. Collaborative Culture**	**2.80** (±1.17)	0.071	−0.869	*t*: 37.962; *p < 0*.001
7. Formative assessment	3.21 (±1.02)	−0.002	−0.519	*t*: 49.895; *p < 0*.001
8. Digital technology	3.28 (±1.044)	0.020	−0.492	*t*: 49.974; *p=0*.001
9. Social engagement (SE)	3.00 (±1.25)	−0.052	−0.994	*t*: 38.210; *p < 0*.001
General rubric of teaching based on socioformative projects	3.156 (±1.43)	0.101	−0.581	*t*: 40.798; *p < 0*.001

Note: Sig. t-test, p< 0.001; distribution ranges: μ + 1σ = 3.372 and μ - 1σ = 2.940.

∗Very high and very low values.

The values in bold were the highest and lowest in each of the teaching based on socioformative projects addressed.

Regarding learning strategies, Table 4 shows the degree of application of these strategies by the students. In this regard, it can be observed that only the class participation, academic communication and the self-evaluation obtained significant scores, above 3.0 (theoretical mean that corresponds to the acceptable or medium level). The same occurred with the overall rubric measure of all learning strategies, which was above the theoretical mean. However, no measure reached the medium high or high level. It should also be noted that there was one strategy that presented a value below the expected mean, and that was Information Search, although the difference was not significant, but it was in the range of the mean plus or minus the statistical deviation. Finally, the overall measure of the rubric learning strategies and the class participation had a small effect size (r= 0.10 - < 0.3); self-evaluation, a moderate effect size (r= 0.30 - < 0.5) and the academic communication a large effect size (r > 0.5).

**Table 4:** Degree of application of learning strategies (LE) according to a theoretical value of 3.0 (medium level).

Learning strategies (LS)	Mean (± standard deviation)	V	p	Effect size (*r*)
**1. Class Participation**	**3.179 (1.00)**	**6600**	**0.006∗**	**0.247**
2. Information Search	2.956 (0.90)	4665	0.450	0.068
**3. Academic Communication**	**3.440∗** (1.05)	10548	0.001∗∗	**0.504**
4. Digital Technology	3.048 (0.96)	6369	0.350	0.081
5. Class Preparation	3.036 (0.97)	4869	0.52	0.061
6. Problem Solving	3.036 (0.98)	6502	0.470	0.062
7. Projects with Social Impact	3.008 (0.96)	5306	0.860	0.017
8. Teamwork	3.060 (1.24)	5718	0.92	0.010
9**. Self-evaluation**	**3.238** (1.052)	6188	0.001∗∗	**0.328**
**Overall measure of the rubric learning strategies**	**3.111 (0.725)**	16208	0.03∗	**0.169**

Note: Wilcoxon ∗Sig., p< 0.05; ∗∗Sig., p< 0.001; distribution ranges: μ + 1σ = 3.253 and μ - 1σ = 2.966.

∗Very high and very low values.

Bolded values had significant differences.

### 4.3 Analysis of the relevance of online activities in face-to-face university programs

In relation to the relevance of online training as a complement to face-to-face university programs, Table 5 presents the results. In this regard, it can be observed that all the variables related to the relevance of this process were significantly above the expected theoretical mean of 3.0 (acceptable level). However, no variable reached high or very high levels. The aspect best evaluated by the students is that the **Virtual Classroom Interaction** (virtual classroom has interactive activities and resources to enhance learning).

**Table 5:** Degree of relevance of online training as a complement to on-site university programs.

Variable	Mean (±SD)	Asymmetry	Kurtosis	*t*-test (Theoretical mean: 3.0)
1. Overall Satisfaction with Online Learning. Assessment of the e-learning platform, content, and online learning activities.	3.12 (±0.22)	−0.167	−0.111	*t*: 14.01; *p < 0*.001
2. Degree of Usefulness of Online Activities. Practical value and applicability of activities conducted in the virtual environment.	3.11 (±0.94)	−0.0622	−0.234	*t*: 1.34; *p < 0*.001
3. Virtual Classroom Friendliness. Ease of use, navigation, and interaction within the online learning platform.	3.23 (±2.033)	−0.011	−0.333	*t*: 1.44; *p=0*.001
4. Virtual Classroom Organization. Logical and coherent structure of the content, activities, and resources in the virtual environment, including clarity of instructions, sequencing of learning, and arrangement of educational elements.	3.24 (±0.95)	−0.0222	−0.52	*t*: 2.333; *p < 0*.001
**5. Virtual Classroom Interaction. Extent to which the e-learning platform facilitates participation in collaborative activities, discussion forums, and feedback spaces.**	**3.56∗**(+0.93)	−0.0111	−0.234	*t*: 2.333; *p < 0*.001
6. Degree of Attraction. Level of motivation and interest that the e-learning platform generates for users to engage and remain active within its environment.	3.12 (±0.22)	−0.167	−0.111	*t*: 14.01; *p < 0*.001
7. Adaptive Classroom. Capability of the virtual environment to adapt to the individual learning needs, preferences, and paces of students, including content customization.	3.13 (±0.22)	−0.167	−0.111	*t*: 14.01; *p < 0*.001

Note: Sig. t-test, p< 0.001; distribution ranges: μ + 1σ = 3.377 and μ - 1σ = 3.054.

∗Value very high.

The value in bold was the highest.

Regarding the degree of satisfaction with online activities and resources in on-site university programs, Table 6 describes this as a percentage. The following can be observed: the activity with the highest acceptance is interactive videos, essentially in the last semesters, and the second most preferred resource is short videos, fundamentally in the first semesters.

**Table 6:** Percentage of satisfaction and relevance of online learning activities and resources in support of face-to-face education.

Online learning activities and resources	Percentage of preference	Semesters 1-4	Semesters 5-7	Semester 8 or higher
Studying long videos (more than 4 min long)	1.587 %	0.794 %	0.794 %	0 %
Participation by WhatsApp	3.175 %	2.381 %	0.000 %	0.794 %
Studying short videos (less than 4 min long)	**10.319 %**	5.556 %	1.588 %	3.175 %
Study interactive videos in which you can participate by answering questions or making comments.	**13.492 %**	4.365 %	2.778 %	6.349 %
Study PDF documents	6.55 %	2.77 %	1.55 %	2.23 %
Performance of multiple-choice tests	1.588 %	0.000 %	0.000 %	1.588 %
Conducting case analysis	4.366 %	1.191 %	0.397 %	2.778 %
Participation in forums	7.144 %	3.572 %	1.191 %	2.381 %
Participation in chat sessions (without video)	4.762 %	2.380 %	0.794 %	1.588 %
Participation in tele-classes with teachers	8.730 %	4.365 %	1.587 %	2.778 %
Participation in online games	0.794 %	0.00 %	0.00 %	0.794 %
Information consultation activities	1.985 %	0.00 %	0.397 %	1.588 %
Assigned application tasks	5.952 %	5.158 %	0.397 %	0.397 %
Study mind maps or infographics	6.350 %	1.985 %	1.190 %	3.175 %
Video classes recorded by teachers uploaded to the platform	5.507 %	1.158 %	1.794 %	2.555 %
Have e-mail communication	3.176 %	1.588 %	1.191 %	0.397 %
Readings on web pages or blogs	4.762 %	2.381 %	0.397 %	1.984 %

On the other hand, with respect to the least valued or least motivating learning activities for students, we find (Table 7): studying long videos (15.66 %) and participating in chat sessions (11.89 %), especially at the end of the careers. A close value was participation by WhatsApp (11.51 %), also at the end of the careers. A remarkable fact was that no student pointed out interactive videos as demotivating or not very relevant.

**Table 7:** Percentage of online activities and resources less motivating for students in face-to-face programs.

Activities and resources	Percentage of dissatisfaction	Semesters 1-4	Semesters 5-7	Semester 8 or higher
Studying long videos	**15.66 %**	5.85 %	3.58 %	6.23 %
Participation by WhatsApp	**11.51 %**	3.96 %	2.08 %	5.47 %
Study short videos	3.02 %	1.32 %	0.38 %	1.32 %
Study interactive videos in which you can participate by answering questions or making comments.	0.00 %	0.00 %	0.00 %	0.00 %
Study PDF documents	10.19 %	6.04 %	1.89 %	2.26 %
Perform multiple-choice tests	3.77 %	1.13 %	0.38 %	2.26 %
Conduct case analysis	3.96 %	2.26 %	0.00 %	1.70 %
Participate in forums	4.15 %	2.26 %	0.57 %	1.32 %
Participate in chat sessions (no video)	11.89 %	4.53 %	2.45 %	4.91 %
Participate in teleclasses with teachers	6.04 %	2.26 %	1.32 %	2.45.%
Participate in online games	7.74 %	3.21 %	1.13 %	3.40 %
Information consultation activities	2.26 %	1.32 %	0.38 %	0.57 %
Assigned application tasks	5.47 %	3.02 %	0.94 %	1.51 %
Study mind maps or infographics	2.45 %	1.13 %	0.57 %	0.75 %
Video classes recorded by teachers uploaded to the platform	4.72 %	2.26 %	1.55 %	0.94 %
Readings on web pages or blogs	7.17 %	3.77 %	0.19 %	3.21 %

### 4.4 Variables associated with the development of transversal skills in face-to-face university programs with online activities

Table 8 presents the regression analysis for to determine the variables that are predictors of the degree of development of the transversal skills. In this regard, for the general measure of transversal skills, the predictor variables were: General measure of learning strategies, semester performance score, general measure of teaching based on socioformative projects (TSP-9), inclusion (TSP-9 variable) and problem solving. The highest power of association was for the variable General measure of learning strategies (β = 0.761, p < 0.001). In the same Table 8, the other variables associated with the specific transversal skills can be seen.

**Table 8:** Linear regression on the association of teaching based on socioformative projects and online education activities with the level of development of transversal skills.

Dependent variable	Predictor variable	C. non-standardized B (Error)	C. standardized Beta	t (Sig.)	Collinearity statistics	
					Tolerance	VIF
General measure TS-10	(Constant)	2.224 (0.180)		12.364 (0.001)		
	Overall measure of the learning strategies rubric	0.070 (0.008)	0.761	9.323 (0.001)	0.241	4.148
	Semester performance score	0.008 (0.001)	0.515	6.400 (0.001)	0.248	4.026
	Overall measurement of TSP-9	0.389 (0.071)	0.540	5.513 (0.001)	0.168	5.968
	Inclusion (TSP-9)	0.117 (0.033)	0.231	3.512 (0.001)	0.373	2.684
	Problem solving (LS)	0.100 (0.038)	0.160	2.630 (0.009)	0.432	2.315
Digital technology	(Constant)	1.585 (0.221)		7.161 (0.001)		
	Overall measure of the learning strategies rubric	0.046 (0.013)	0.395	3.543 (0.001)	0.300	3.336
	Problem solving (LS)	0.158 (0.073)	0.200	2.161 (0.032)	0.433	2.308
	Academic communication (LS)	0.134 (0.068)	0.162	1.979 (0.049)	0.557	1.795
Social-emotional skills	(Constant)	3.087 (0.281)		10.977 (0.001)		
	Collaborative culture (TSP-9)	0.198 (0.058)	0.222	3.420 (0.001)	0.804	1.244
	Interactive and dynamic activities (TSP-9)	0.334 (0.102)	0.291	3.284 (0.001)	0.431	2.318
Collaborative work	(Constant)	1.709 (0.377)		4.540 (0.001)		
	Collaborative culture (TST-9)	0.150 (0.073)	0.162	2.048 (0.042)	0.432	2.315
	Student management (TST-9)	0.149 (0.066)	0.155	2.250 (0.025)	0.570	1.755
	Overall measure of the learning strategies rubric	0.032 (0.015)	0.176	2.110 (0.036)	0.389	2.572
Management of continuous training	(Constant)	2.562 (0.423)		6.062 (0.001)		
	Overall measure of the learning strategies rubric	0.924 (0.158)	0.653	5.849 (0.001)	0.243	4.107
	Overall measurement TSP-9	0.454 (0.126)	0.369	3.614 (0.001)	0.292	3.430
	Degree of attraction	0.218 (0.070)	0.207	3.104 (0.002)	0.680	1.471
	Virtual classroom interaction	0.181 (0.067)	0.175	2.684 (0.008)	0.713	1.402
	Problem solving (LS)	0.212 (0.089)	0.200	2.384 (0.018)	0.432	2.317
Complex thinking	(Constant)	3.036 (0.280)		10.857 (0.001)		
	Overall measure of the learning strategies rubric	0.585 (0.094)	0.507	6.233 (0.001)	0.397	2.520
	Adaptive classroom	0.136 (0.047)	0.185	2.926 (0.004)	0.654	1.530
	Collaborative culture (TSP-9)	0.143 (0.049)	0.203	2.899 (0.004)	0.537	1.862
	Formative assessment (TSP-9)	0.133 (0.058)	0.167	2.284 (0.023)	0.493	2.028
Leadership	(Constant)	1.979 (0.319)		6.208 (0.001)		
	Overall measure of the learning strategies rubric	0.476 (0.106)	0.325	4.498 (0.001)	0.666	1.502
Research	(Constant)	2.614 (0.290)		8.998 (0.001)		
	Overall measure of the learning strategies rubric	0.186 (0.084)	0.168	2.214 (0.028)	0.633	1.580
Oral and written communication	(Constant)	0.712 (0.273)		2.614 (0.010)		
	Overall measure of the learning strategies rubric	0.040 (0.014)	0.264	2.887 (0.004)	0.266	3.759
	Academic communication (LS)	0.185 (0.070)	0.193	2.641 (0.009)	0.416	2.406
	Academic communication (LS)	0.146 (0.066)	0.144	2.229 (0.027)	0.534	1.874
	Student management (TSP-9)	0.193 (0.052)	0.239	3.754 (0.001)	0.548	1.823
	Inclusion (TSP-9)	0.153 (0.056)	0.183	2.707 (0.007)	0.484	2.065
	Interactive and Dynamic Activities (TSP-9)	0.225 (0.062)	0.226	3.650 (0.001)	0.576	1.735
	Collaborative culture (TSP-9)	0.122 (0.051)	0.145	2.391 (0.018)	0.600	1.667
	Virtual classroom organization	−0.207 (0.067)	−0.196	−3.112 (0.002)	0.557	1.794
	Online study satisfaction	0.163 (0.063)	0.163	2.602 (0.010)	0.562	1.778
Entrepreneurship	(Constant)	1.419 (0.318)		4.466 (0.001)		
	Overall measure of the learning strategies rubric	0.445 (0.097)	0.316	4.565 (0.001)	0.640	1.561
	Collaborative culture (TSP-9)	0.173 (0.056)	0.214	3.071 (0.002)	0.632	1.583
English	(Constant)	1.088 (0.259)		4.198 (0.001)		
	Academic communication (LS)	0.244 (0.083)	0.210	2.924 (0.004)	0.784	1.275

### 4.5 Variables associated with the academic performance of students in face-to-face university programs with virtual activities

**Fig. 2:**
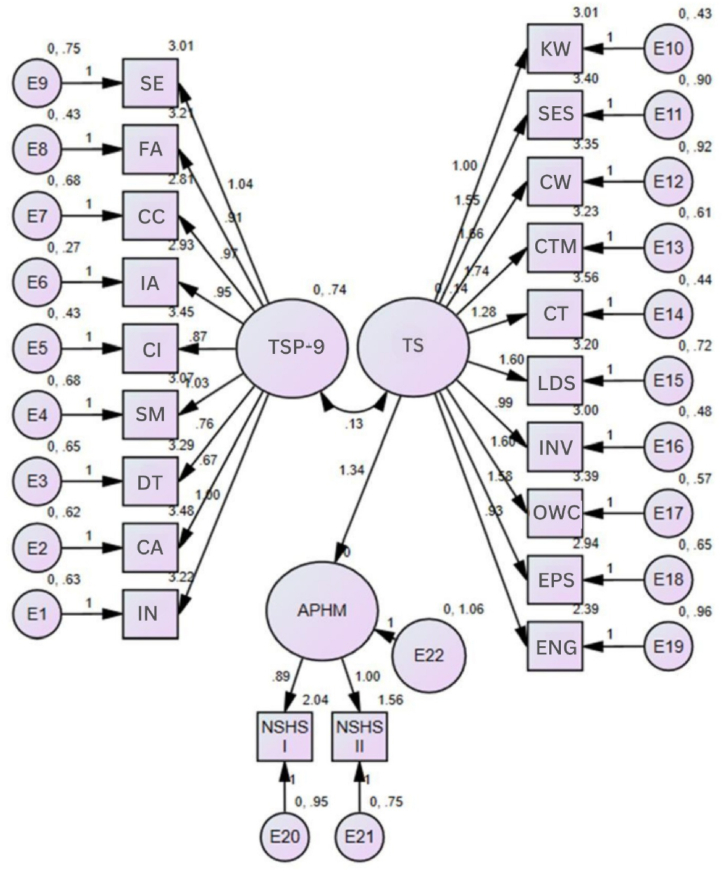
Standardized solution of the proposed model. *Note*. TSP-9=Teaching based on socioformative projects; SE=Social Engagement; FA=Formative assessment; CC= Collaborative Culture; IA=Interactive and Dynamic Activities; CI=Community Issues; SM=Student Management; DT=Digital Technology; CA=Critical Analysis; IN=Inclusion. TS= transversal skills, KM= knowledge management, SES= socioemotional skills, CW= collaborative work, CTM= continuous training management, CT= complex thinking, LID= leadership, IN= investigation, OWC= oral and written communication, EPS= entrepreneurship, ENG= English, APHM= academic performance with high score, NSHS I= number of subjects with a high score in the previous semester; NSHS II= number of subjects with a high score during the current semester.

The following is the regression analysis to determine the factors associated with the academic performance obtained by students in the previous semester and the current semester, in on-site university programs with virtual activities. In this regard, it can be established that in all academic performance variables there is an association with the general measure of transversal skills or a specific skill (Table 9) (see Fig. 2).

**Table 9:** Multiple linear regression with respect to academic performance variables in students of face-to-face university programs with virtual activities.

Dependent variable	Predictor variable	Unstandardized coefficients	Standardized coefficients	t (Sig.)	95 % CI for B		Collinearity statistics	
		B (Desv Error)	Beta		Lower limit	Upper limit	Tolerance	VIF
Academic performance in the current semester	Constant	−122.347 (6.815)		−17.952 (0.001)	−135.778	−108.916		
	Overall measurement of TSP-9	3.095 (0.286)	0.619	10.808 (0.001)	2.531	3.659	2.99	3.341
	Overall size of the TS-10	1.933 (0.210)	0.310	9.223 (0.001)	1.520	2.346	0.872	1.146
	Interactive and dynamic activities (TSP-9)	4.816 (2.186)	0.127	2.203 (0.029)	0.507	9.124	0.297	3.368
Previous semester's grades	Continuous training (TS-10)	1.413 (0.596)	0.157	2.369 (0.019)	0.238	2.588	1.000	1.000
Number of subjects with a high grade in the previous semester	Constant	−1.517 (0.512)		−2.963 (0.003)	−2.525	−0.508		
	General measure TS-10	0.406 (0.163)	0.172	2.493 (0.013)	0.085	0.728	0.724	1.380
	Semester in progress	0.131 (0.026)	0.300	5.028 (0.001)	0.080	0.182	0.963	1.038
	Academic communication (LS)	0.355 (0.104)	0.230	3.401 (0.001)	0.001	0.149	0.560	0.752
Number of subjects with high grades in the current semester	Constant	−0.689 (0.477)		−1.436 (0.002)	−1.626	0.255		
	General measure TS-10	0.062 (0.020)	0.252	3.110 (0.001)	−0.023	0.101	0.521	1.921
	Overall measure of the learning strategies rubric.	0.348 (0.166)	0.175	2.090 (0.038)	0.020	0.675	0.484	2.068
Number of subjects not passed in the previous semester	Constant	0.868 (0.507)		1.710 (0.089)	−0.132	1.868		
	Social-emotional skills (TS-10)	−0.380 (0.298)	−0.320	−4.780 (0.001)	−0.455	−0.141	0.640	1.562
Suspension of studies during the course of study	Constant	0.809 (0.234)		3.451 (0.001)	−0.347	1.271		
	Adaptive classroom	−0.130 (0.050)	−0.172	−2.599 (0.010)	−0.229	−0.031	0.954	1.048
	Collaborative culture (TSP-9)	−0.124 (0.050)	−0.168	−2.465 (0.014)	−0.223	−0.033	0.954	1.048

Additionally, it was proven through structural equation modeling (SEM), that teaching based on socioformative projects and transversal skills are significantly correlated (p < 0.001) with direct effects on each other. In addition, transversal skills were found to have an effect on academic performance variables, such as the number of subjects passed with high grade in the previous semester and the current semester (p < 0.001). The proposed model is illustrated in Fig. 2 and the goodness-of-fit indices are detailed in Table 10, which were adequate.

**Table 10:** Model adjustment.

Statistician	Reference value	Value obtained	Decision
χ2	–	218	Acceptance
gl	–	186	Acceptance
Ratio χ2/gl	<3.0	1.175	Acceptance
IFC	>0.95	0.995	Acceptance
TLI	>0.95	0.994	Acceptance
GFI	>0.95	0.981	Acceptance
NFI	>0.95	0.965	Acceptance
PNFI	Close to 1	0.854	Acceptance
RMSEA	<0.08	0.026	Acceptance

## 5 Discussion

### 5.1 Level of development of transversal skills in face-to-face university programs with online activities

There is evidence that transversal skills such as communication and teamwork are essential for employability in the current context and will be fundamental in the near future [[77], [78], [79], [80]]. Even in areas considered highly technical, where specific skills play a central role, transversal skills have been found to be essential, such as communication, which is the most prominent, followed by proactivity and teamwork, but also information and communication technologies [81]. However, various studies suggest that higher education still has difficulties and failures in forming the transversal skills required by the context [81]. Therefore, it is recommended to implement actions for their evaluation and development, based on relevant didactic strategies [82]. In the present research, contrary to other studies, transversal skills, taken as a general measure, have a development at a medium level, which is acceptable because it meets the minimum expected actions; and, if addressed separately, most transversal skills (seven) have a level above the mean, although not at a high or very high level.

In the present research (Table 2), the transversal skills with the highest development and application in students was complex thinking, without reaching a medium-high or high level. This data is important because it has been found that complex thinking skills, such as analysis and problem-solving, creativity, innovation, adaptation, and flexibility, are among the aspects most demanded by employers and are highly valued by recent graduates of university programs [83]. In some university training programs, it has been found that there is a need to further develop critical thinking [84,85], which is another essential axis of complex thinking, due to a lack of more teacher training or not having enough time to do it within the study plans. However, there are reports that in higher education, critical thinking and analytical ability have good development [12,[86], [87], [88]], and this has also been found in online or virtual university programs [89]. These processes are individual in nature, not involving the interpersonal or collective. This may be the result of applying the pedagogical model of constructivism in virtual education, which is centered on each student's learning [90]. However, a contribution of the present study is that complex thinking has been evaluated considering its various dimensions based on solving real-world problems by articulating diverse knowledge with flexibility, to enable solutions that help transform communities and organizations, as socioformation seeks. Furthermore, the results show that it is the most developed competency in face-to-face programs complemented with online training.

Socioemotional skills consist of processes aimed at managing emotions so that they do not affect people and help them in their daily activities. These skills are considered essential in training activities, and it is recommended that universities strengthen them in academic programs [91]. Despite the insistence in various works and documents that the pandemic generated various socioemotional problems in students [92], and that the level of development of socioemotional skills tends to be perceived as low in higher education, in the present study the transversal practice of socioemotional skills had a medium or acceptable development, which can be a strength to continue studies after the COVID-19 pandemic.

The present research also found an acceptable or medium level of development of transversal skills in communication and digital technology, which are related. Regarding communication, previous studies have found an acceptable level [89] and a low level in students regarding text comprehension [93] and critical reading [11]. Regarding digital technology, it is an essential competency that should be formed in the university with specific strategies, but a deeper training in students is lacking because it is often reduced to using digital applications without evaluation [94]. Contrary to the present findings, previous studies found significant deficits in this transversal competency [12], which may be due to a poorly relevant and critical use of technology [95]. This same result has been observed in online education [89,96]. It is possible that in the present study, students have greater development of digital competency due to the performance of online activities following a more problem-oriented pedagogical model. Digital technology-oriented thinking could be improved with the establishment of professional networks among students and the enhancement of informal learning [97], which can be done with online education, complementing face-to-face education.

Close to complex thinking is the transversal practice of continuous training, which is considered fundamental to face challenges and the future in the professional field and is defined as the process by which people learn knowledge to address needs, such as those derived from employment [98]. In the present research, it was found that this practice presents a medium level of development in students, occupying the fifth place based on its mean (Table 2). In previous studies, an acceptable level of development of skills related to continuous training has been found, such as autonomy [89].

Although collaboration is an essential competency for living in society and is one of the skills most demanded by companies [99], several studies have found that university students perceive a low level in its development, or at least lower than minimally expected [88,[100], [101], [102], [103]], and this has also been found in the virtual study modality in higher education [89]. In the present research, on the contrary, it was found that collaborative work has a medium or acceptable level, which may be the result of the pedagogical model centered on the interactive classroom that is used in the university. This is in line with other studies [12,104], and shows that transversal skills can be developed when addressed through face-to-face activities complemented with online activities [105], based on addressing problems in the environment.

Leadership, for its part, is conceived as an essential practice for professionals to coordinate processes of improvement, change, and innovation in the contexts in which they operate, through collaboration with other people. In some university programs, it has been found that this practice does not have sufficient development in students [84], probably because more training and time are lacking within the study plans. In the present research, leadership had an acceptable development and was the sixth practice with better development in students, within the framework of face-to-face training complemented with online education.

It is important to note that the seven transversal skills described had an acceptable or medium level of development within the framework of a strong change in the study modality, as students moved from 100 % face-to-face education to having complementary virtual activities, as a result of the experience lived in the pandemic that helped generate this change. The fact of having obtained these good results could be explained by several reasons: 1) the implemented online education platform, which, being structured through different activities and interactive resources, and having good satisfaction from students, enabled these levels; or 2) the fact of previously having a quality face-to-face training system, given that it is a public university with good levels of educational impact, which has a student-centered pedagogical model and aims for inclusion [106].

Studies on the level of development of transversal skills in higher education are discrepant or contradictory. Some indicate that there is a low degree of development in students [107], while other investigations have found the opposite, that their development is acceptable [11,32]. The present research provides evidence of the latter, as most of the evaluated transversal skills had a relevant development, and the general measure of these competencies had an acceptable level, which shows that the option of articulating face-to-face studies with virtual activities can be relevant. Only three competencies had a level below the mean: English, research, and entrepreneurship. English is essential for the labor world and employability in Latin America, although in other contexts it may not be [108], and in the present study, it was the practice with a significantly lower level. This is similar to what has been found in other studies in Latin American universities, in which English has a low level in students [109]. Regarding entrepreneurship, this is a competency little addressed in higher education, and in the university where the present study was conducted, it is not explicitly in the curriculum, which may explain why the level of this competency was low in students. There is evidence that when entrepreneurship is worked on with specific actions within the curriculum, students strengthen their entrepreneurial spirit [110]. Something similar happened with the transversal competency of research, which was also low in students, possibly due to the lack of work on it in the educational model.

### 5.2 Teaching based on socioformative projects and learning strategies

In the realm of higher education, understanding the factors that influence the relevance of on-campus university programs with online education components has become increasingly crucial. To address this issue, a comprehensive analysis of teaching based on socioformative projects and learning strategies was conducted from the perspective of the socioformation pedagogical model. The results of this analysis revealed that the overall implementation of teaching practices based on socioformative projects reached an acceptable level, as evidenced in Table 3. A more detailed examination of pedagogical practices centered on these projects also showed a medium or acceptable level, surpassing the theoretical mean of 3.0, which is consistent with findings from previous research [111,112]. These results are indicative of significant progress in the adoption of methodologies based on socioformative projects in higher education, reflecting a growing trend towards incorporating real problems and a more transversal approach in universities [113]. It is important to note that this advancement has occurred in a university context where the methodology of socioformative projects has not yet been explicitly established as a central strategy in educational programs.

Online education has proven to be an important catalyst in promoting more environmentally-oriented professional training [114]. From the socioformation perspective, this approach materializes through the active engagement of universities with their surrounding communities. The main objective is to foster, from the curriculum itself, transformation processes that contribute to improving living conditions and promoting environmental sustainability. This approach necessarily implies the adoption of interdisciplinary practices and addressing real-world problems, aspects that are facilitated and enhanced by the integration of virtual education [115]. In response to this trend, some higher education institutions have begun to innovate their online training processes. A notable example is the Open University of the United Kingdom, which has implemented multidisciplinary modules specifically designed to develop essential transversal skills such as problem-solving, effective communication, collaboration, and global citizenship, among others [116]. These initiatives represent a significant step towards curricular and didactic change in universities to focus more on societal transformation and train creative and innovative professionals, aiming for them to be more than just centers that adapt to environmental demands.

Nevertheless, the present research identified significant areas of opportunity in the implementation of socioformative projects, particularly regarding two fundamental pedagogical practices: interactive and dynamic activities, and collaborative work culture. These aspects showed a lower-than-expected level of application in students' educational experiences, a trend that seems to have been accentuated during the pandemic period. Parallel studies have corroborated this observation, noting a notable decline in interaction between teachers and students in online educational settings during the health crisis, as well as a decrease in positive feedback from teachers [117]. It is plausible that the challenges imposed by the abrupt transition to online education have contributed to this reduction in interactive and collaborative activities, an aspect that has not yet fully recovered. Existing literature emphasizes the critical need to enhance interactive elements in teaching [118], highlighting the potential of social media as ideal instruments to foster interactive learning that can also help mitigate student anxiety towards complex academic content. The generational gap between professors and students could be an underlying factor in the low level of interactive activities observed in the present study, suggesting a possible difficulty for teachers in adapting their methodologies to the preferences and learning styles of new generations. Addressing this gap and promoting the implementation of more interactive and participatory activities, leveraging digital technology, social media, and short video formats, presents itself as an urgent need in contemporary higher education [119].

It is important to contextualize these findings within the broader framework of research on pedagogical practices in higher education. While previous studies [120,121] have explored pedagogical practices related to the development of transversal skills, these have primarily focused on conceptualizing them as teaching skills or as activities inherent to the teaching process. In contrast, the present research adopts a distinct approach by examining pedagogical practices from the perspective of socioformation. Under this new pedagogical model, socioformative projects are conceived as performances or actions specifically oriented towards training students in solving contextual problems, with the ultimate goal of contributing to sustainable social development. This approach represents a significant contribution to the field, given that studies addressing pedagogical practices from this holistic and socially-oriented perspective are relatively scarce. Socioformation, by emphasizing the link between academic learning and real societal challenges, provides a valuable framework for rethinking and redesigning educational practices in higher education, aligning them more closely with the needs of a constantly evolving world and the demands of global sustainability.

An element associated with pedagogical practices from the socioformation perspective is the use of learning strategies to enhance problem-solving through the appropriation of diverse knowledge. In this regard, the present research found that the general measure of learning strategies was at a medium level, and three specific strategies presented an application above the theoretical mean, without reaching the medium-high or high levels (Table 4): class participation, academic communication, and self-evaluation. Furthermore, learning strategies were predictors of better academic performance. A recent study found that learning strategies were predictors of greater academic success [122], but without establishing which performance variables they directly affect. What the present research contributes is information about the aspect of academic performance through which final success can be achieved, which could be through achieving subjects with high grades.

### 5.3 Satisfaction and relevance of online education activities, in complement to face-to-face training in university programs

There are reports showing that during the COVID-19 pandemic, students had low satisfaction with the online education that had to be implemented as a consequence of school closures [123,124] and in other cases an ambivalent position on this modality, that is, with positive and negative assessments [125]. This may be due to factors such as lack of face-to-face interaction and dialogue with teachers and classmates [122], excess homework [124,125], and difficulties in concentration [125], within an educational context with deficiencies in the organization of activities and lack of more collaboration among students [123,126,127], resulting from educational models centered on content, as well as the absence of attractive and motivating virtual classrooms that generate interaction among students [123,128].

Unlike these previous studies that show dissatisfaction with virtual education during the COVID-19 pandemic, the present research clearly found that students are satisfied at a medium or acceptable level with this study modality at the University where the research was conducted, in all the variables considered (Table 5), such as satisfaction, resources, adaptation, relevance, etc. This means that the University managed to get students to assume virtual education as a relevant process, which is a purpose in current higher education [53], and that the activities were relevant and with interaction (the latter is supported by the fact that the best-valued action of the e-learning platform was having interactive activities in virtual classrooms) [129].

These positive results can be explained by the fact that the online education implemented in this university as a complement to face-to-face programs integrated socioformation principles, such as the following: 1) adaptive virtual classroom, flexible to changes and student needs; 2) inclusion in learning activities and resources; 3) motivating and attractive resources; and 4) interactivity. Having a clear pedagogical model in virtual education helps e-learning work to have better results due to better organization of activities, better articulation of technology, and increased relevance of training actions considering the student. In other experiences, it has already been determined that a solid pedagogical model favors virtual learning in students, as found in the Universidade Aberta (UAb) [90]. In addition, it is known that the use of digital technology is related to better educational processes [42], as well as having an online platform as support [130], and that videos are associated with greater development of critical thinking [131], which could help explain the results obtained. Likewise, it has been found that information and communication technologies, such as those used in virtual education, are associated with better social circles [132].

There is evidence that digital resources are essential in learning in online education, so it is necessary to strengthen them [133]. However, information is lacking on which digital resources are priority. In this regard, the present study provides evidence that the digital resources best valued by students are interactive videos and short videos of less than 4 min (Table 6), which is in line with social trends in the technological sphere, such as the strong positioning of short videos on TikTok or YouTube. In previous studies, this was already beginning to be visualized. For example, in the research by Alonso-García et al. [134], it was found that students preferred presentations and teacher videos, as well as videoconferences. However, the present research provides new elements: not just any video is relevant, they should be short, and, if possible, interactive, that is, seeking student participation by answering questions, sharing reflections, or seeking complementary information, from the video itself. Therefore, interactivity is a relevant element in current online education [129] that should continue to be strengthened.

Regarding the least motivating or relevant virtual resources or activities for students, the present research shows that they are long videos and participating in chat sessions (Table 7). This partially agrees with the study by Alonso-García et al. [134], in which it was found that chat sessions were among the least motivating or preferred for university students during the pandemic, along with forums and bibliography. An important difference with other previous studies [134,135] is that in the present research, videoconferences were not among the preferences of students, which may be due to their excess, high duration, or lack of a motivating and participatory didactic methodology.

Do technological tools help to have better academic performance? In the present research, online education components, such as having an adaptive virtual classroom, mainly helped to decrease dropout rates, but did not have implications for academic performance. In a previous study, digital resources were associated with greater subject approval [136]. The positive relationship of the virtual classroom with lower dropout rates would be sufficient to justify the incorporation of this complement in face-to-face university programs. However, there was also an association of several aspects of complementary virtual education with greater development of transversal skills, such as the e-learning platform, the learning system, the organized virtual classroom, and satisfaction with online study. The latter was also reported by a recent investigation in which online education in the last year of a nursing career was associated with better transversal skills and better preparation for the professional world [130].

### 5.4 Factors associated with the development and application of transversal skills

What factors are associated with the development and application of transversal skills? The present study provides evidence that teaching based on socioformative projects, learning strategies, and online education are associated with the development and use of transversal skills [15] (Table 8). This may be due to the change in educational modality, which led university teachers to the need to train in the use of digital technologies, as well as the incorporation of more participatory didactic strategies, more oriented to society's problems, as is done in socioformation. In this regard, there is evidence that participation in community service activities is associated with greater development of generic skills [137], as well as the use of sociodramas [138].

Some studies have demonstrated that learning strategies linked to new technologies and collaborative work are highly relevant to ensure that students can have a space for significant learning and with great motivation. In this study [139], a test with immersive virtual reality was conducted where the above could be verified, especially that in interactive digital spaces, collaborative work tends to be motivating for students; however, the authors recommend channeling activities towards the learning objective. And contrasting to this is the study that focuses on students' perception of practices, where they state that to acquire comprehensive knowledge that can be brought to society, it is necessary to have the combination of theoretical knowledge and problem-centered real-world practices [140]. These same authors demonstrate in their study how research internships that include transversal skills that include academic knowledge and laboratory practices are of fundamental importance to produce knowledge that is useful for society.

### 5.5 Association of transversal skills with academic performance

In the present research, it was found that a higher level of development and application of transversal skills (taken as a general measure) was associated with better academic performance in the semester and a higher number of subjects with high grades (Table 9). In addition, it was found that continuous training was associated with better semester grades, and socioemotional skills were related to greater subject approval. It is possible that this is due to the articulation with teaching based on socioformative projects, as shown by the structural equation model.

In the present study, no association was found between complex thinking and any of the academic performance variables, such as average, subject approval, recognitions, or dropout. This relates to a recent longitudinal study in which no association was found between problem-solving and inductive reasoning (aspects integrated within complex thinking in the present research) with academic success [122]. The possible explanation is that complex thinking is still not relevant in subjects because they focus more on content, but it is feasible that it is key to professional success when students graduate. The same happened with the digital technology competency, despite a previous study finding an association of this competency with lower subject repetition rates [136]. It has also been reported that collaborative work is associated with better academic results in online education activities [133]. However, the role of the pedagogical practice of teacher-mediated collaboration in this process has not been investigated. The present research provides evidence that a culture of collaborative work guided by teachers has positive effects on academic success, helping to reduce dropout rates in university studies.

There is evidence that the use of digital technologies and virtual education based on solid pedagogical models or strategies, during the COVID-19 pandemic, helped to achieve the expected learning outcomes in the curriculum and strengthened the integral formation of students [141]. This is similar to what was reported by Ref. [142], regarding the effects of hybrid education during the pandemic, in the sense that these practices can be associated with more relevant educational processes. Accordingly, in unfavorable situations, it has been found that teachers and students can adjust to situations by establishing a work path, and thus obtain acceptable learning results and not reach study abandonment [143,144].

### 5.6 Use of rubrics in the evaluation of transversal skills

In the present research, scales were not used to evaluate transversal skills, but rather analytical rubrics, which possibly have greater reliability [145,146], because they evaluate the different elements through concrete descriptors. Generic competencies have been diagnosed in most cases through Likert-type scales, which possibly have less reliability for being more subjective in the way of evaluation by lacking descriptors [147,148], which are present in rubrics. Furthermore, the use of rubrics possibly helps better self-evaluation and learning, as evidence has been provided that it helps the development of generic competencies, such as reflective writing [149]. In addition, in the present study, rubrics were used from a new perspective, such as socioformation, which are based on a new taxonomy [8], which is of Latin American origin, and emphasize addressing problems of the territory, not content, as is the traditional methodology. This new rubric methodology helps to assess and improve the transformation of living conditions in communities, based on integral actions that articulate values with skills.

This study validated three innovative rubrics developed from a socioformative methodology perspective, which are designed to expand tools for enhancing and innovating teaching practices, contributing to the comprehensive development of students: the Transversal Skills Rubric, the Teaching Based on Socioformative Projects Rubric, and the Socioformative Rubric for Learning Strategies. Complete versions of these tools are available in the appendices in both Spanish and English. These rubrics are crafted to bolster self-assessment and promote the development of critical thinking. Unlike traditional rubrics that focus solely on specific subjects, categories, or indicators, these tools utilize reflection questions that address environmental problems and span both the process and the final product. Each reflective question is assessed across five levels of performance, ranging from the most basic to the most complex and systemic, facilitating a progression that enriches self-evaluation options. The descriptors at each level provide clear criteria for identifying achievements and pinpointing areas for improvement when addressing problems, including detailed elements, aspects, and examples of the evaluated processes and products. These descriptors are organized according to the socioformative taxonomy, which includes five levels: receptivo-mecánico, resolutivo, crítico-analítico, reflexivo y co-creativo. Additionally, each rubric includes detailed instructions to facilitate independent use and enhance the effective implementation of these evaluative tools.

### 5.7 Limitations of the study

The limitations of the present study were: 1) there was no contrast with teachers and managers, regarding teaching based on socioformative projects and the relevance of online education [150]; 2) a longitudinal follow-up was lacking to more clearly establish the predictive power of transversal skills on academic performance in subsequent semesters; 3) the study was based only on student self-evaluation and did not take into account the observation and recording of transversal competency training actions by experts (for example, reports, written works, videos, record of participation in virtual activities, etc.), which would have provided greater validity to the process [95,151]; 4) we believe that the number of transversal skills could also be further reduced to only five or six, in the same line as the "5Cs" key competencies model [152], but with an orientation towards complex thinking and including socioemotional skills and artificial intelligence; and 5) artificial intelligence was not addressed, which should be considered in future research on the subject [153]. This gap is explained because at the time of planning the study, the degree of development and impact of artificial intelligence in education and in the professional context was not available.

### 5.8 Practical implications of the study

The results of this study underscore the urgent need to transform the university educational model towards a socioformative approach. This transformation is fundamental to address complex global challenges and contribute to achieving the Sustainable Development Goals. In the present study, socioformation demonstrated an impact on the development of transversal skills such as complex thinking and socioemotional skills, which are crucial for the current labor market and the transformation of communities, seeking an improvement in living conditions and care for the environment. This requires the implementation of research project methodology linked to social and business aspects, which articulate dialogue between all actors [154]. Unlike models focused solely on individual learning, socioformation emphasizes collaborative resolution of real environmental problems, better preparing students to face multiple social, economic, and environmental challenges [155]. This approach effectively integrates digital technologies and online activities as tools to enhance meaningful and contextualized learning, essential in the post-pandemic era and for the formation of change agents capable of driving sustainable development.

In addition to the above, four additional practical implications are considered: 1) prioritize the use of short interactive videos on online learning platforms, as these resources are preferred by students and can increase their participation and learning [156]; 2) foster pedagogical practices that promote complex thinking, as it has been shown to be the most developed and valued transversal competency in the professional environment [157]; 3) implement continuous evaluation systems that include socioformative rubrics that enable feedback to students on their performance and guide them in solving problems that help improve living conditions in communities [155]; 4) strengthen research, entrepreneurship, and English competencies in universities through specific programs and their transversal integration into the curriculum, based on projects that address real-world problems as they are essential for the region's development and graduates' professional growth [158]; and 5) need to implement hybrid models that combine the best of face-to-face and online education, redesigning curricula with support in adaptive, agile, simple, and interactive learning platforms that integrate artificial intelligence to personalize the educational experience and improve the reach of community interventions [159].

## 6 Conclusions

1. Regarding the first objective of evaluating the level of development of transversal skills in students of on-campus university programs with online activities, it was found that 7 of the 10 skills assessed presented a medium level of development, with complex thinking being the most developed. However, three skills had a low level: research, entrepreneurship, and English, the latter being the least developed. This suggests that while there is progress in the formation of transversal skills, there are still significant areas of opportunity to address.
2. Concerning the second objective of analyzing the implementation of pedagogical practices based on socioformative projects, it was found that most presented a medium level of application, with inclusion and **Community Issues** standing out. Nevertheless, two practices were below the acceptable level: interactive and dynamic activities, and collaborative culture. Therefore, it is recommended to strengthen these aspects in teacher training and curriculum design from the socioformative model.
3. Regarding the third objective of examining the relevance of online activities as a complement to on-campus education, students reported a medium level of satisfaction overall, with the most valued aspect being that the virtual classroom possesses interactive activities and resources. Additionally, short and interactive videos were the most motivating resources, while long videos and chats were the least preferred. This offers valuable guidance for designing attractive and effective virtual environments.
4. In relation to the fourth objective of identifying variables associated with the development of transversal skills, it was found that the main ones were learning strategies, socioformative projects, and some components of online education. This confirms the relevance of solid teaching mediation and appropriate instructional design, which articulates face-to-face and virtual elements based on real problems of the community-territory, to favor the comprehensive formation of students.
5. Finally, regarding the fifth objective of determining the variables associated with academic performance, it was found that transversal skills, especially when considered collectively, were related to a higher average, a greater number of subjects passed with high grades, and a lower dropout rate. Furthermore, practices based on the methodology of socioformative projects also showed a positive effect on academic performance, mediated by transversal skills. This highlights the impact that developing transversal skills has on student success.

## CRediT authorship contribution statement

**Yolanda Guerra-Macías:** Writing – review & editing, Writing – original draft, Validation, Software, Investigation, Formal analysis, Data curation. **Sergio Tobón:** Writing – review & editing, Writing – original draft, Validation, Supervision, Resources, Methodology, Conceptualization.

## Funding

This work was supported by the Centro Universitario CIFE [grant numbers 002-2020].

## Declaration of competing interest

The authors declare that they have no known competing financial interests or personal relationships that could have appeared to influence the work reported in this paper.

## Figures and Tables

**Table:** 

Transversal Skill	Very Low	Low	Medium	Medium High	Very High
**Digital Technology and Knowledge Management** To what extent do you use technology to search for and apply information to solve real-world problems?	I use my phone or social networks to look up basic information without verifying the sources.	I use search engines like Google and basic programs (Word, PowerPoint) to organize information but do not analyze its quality.	I use academic databases and tools like Excel to search for and analyze information related to problems in my environment.	I handle specialized software to analyze complex information and propose solutions to real problems in my field of study.	I use scientific databases to research and solve complex problems. I assist others in using technology to manage information effectively.
Achieved Level					
Achievements and Areas for Improvement					
**Socioemotional Skills** To what extent do you manage your social and emotional skills to address life's challenges?	Difficult situations greatly affect me, and I frequently have conflicts with others.	I try to respect others, but I struggle to control my emotions in stressful situations.	I control my emotions to avoid impacting others and can understand different viewpoints in conflicts.	I effectively manage my emotions to achieve goals and improve coexistence, using techniques like relaxation.	I use my emotional skills to enhance my environment, helping others resolve conflicts and promoting a positive atmosphere.
Achieved Level					
Achievements and Areas for Improvement					
**Collaborative Work** To what extent do you work collaboratively with others to solve environmental problems?	I prefer to work alone, avoiding collaboration even when necessary.	I participate in group work doing the minimum required without taking initiatives.	I actively collaborate in teams, contributing ideas and fulfilling my responsibilities to solve problems.	I strive to improve my performance and that of the team, proposing solutions to emerging issues.	I effectively lead teams, resolving conflicts and motivating others to achieve high-impact goals and tackle challenging problems.
Achieved Level					
Achievements and Areas for Improvement					
**Continuous Learning** To what extent do you seek to educate and train yourself on your own initiative to face environmental problems?	I only carry out mandatory training activities in my studies.	I occasionally attend extra training activities, mainly to improve my resume.	I actively seek learning opportunities to better prepare myself for environmental problems.	I assess my training needs and seek specific training to enhance my skills.	I manage my continuous learning as part of a life plan, and assist others in improving their skills.
Achieved Level					
Achievements and Areas for Improvement					
**Complex Thinking** To what extent do you analyze and solve problems with critical thinking, creativity, and flexibility?	I struggle to adapt my thinking and tend to follow others' opinions.	I can analyze simple problems but find it difficult to see different perspectives.	I approach problems with flexibility and am able to change my mind based on facts and arguments.	I critically evaluate information from various sources and adjust my strategies according to the situation.	I integrate knowledge from various fields to creatively and innovatively solve complex problems.
Achieved Level					
Achievements and Areas for Improvement					
**Leadership** To what extent do you positively influence others to improve your environment?	I identify basic goals but do not take leadership initiatives.	I can plan to achieve goals but struggle to influence others.	I develop plans to solve problems in my community and begin to guide others.	I manage projects that positively impact my environment, promoting inclusion.	I lead significant changes, empowering others to improve living conditions in the community.
Achieved Level					
Achievements and Areas for Improvement					
**Research** To what extent do you participate in the creation of new knowledge through research?	I understand the basic idea of research but have not participated in projects.	I have assisted with simple research tasks, such as conducting surveys.	I have engaged in research activities with peers or professors.	I actively participate in research projects and have presented results at academic events.	I contribute to the publication of scientific articles and lead research projects on relevant problems.
Achieved Level					
Achievements and Areas for Improvement					
**Communication** express your ideas clearly and effectively, orally and in writing?	I have difficulty expressing myself clearly, both speaking and writing.	I can communicate simple ideas but with vocabulary and structure limitations.	I present ideas clearly in presentations and reports on familiar topics.	I present solid and coherent arguments when addressing complex problems.	I communicate ideas persuasively and assist others in improving their communication skills.
Achieved Level					
Achievements and Areas for Improvement					
**Entrepreneurship** To what extent do you identify opportunities and leverage them to create value in your environment?	I only perform assigned activities without seeking new opportunities.	I have ideas for solving problems but have not yet implemented them.	I have participated in initiatives to address needs in my immediate environment.	I lead projects that address relevant problems in my community.	I develop innovative projects with significant impact and promote entrepreneurship in others.
Achieved Level					
Achievements and Areas for Improvement					
**English** What is your level of English?	I know a few words in English and can form very basic sentences. My understanding and expression are extremely limited.	My English level is elementary. I can make and respond to simple questions about everyday situations, but with difficulty and frequent errors.	My English level is basic-intermediate. I understand and interpret commonly used expressions and phrases related to relevant areas of expertise (family, work, studies). I can communicate in simple and routine tasks requiring a simple exchange of information.	My English level is intermediate-advanced. I can interact with native speakers with a sufficient degree of fluency and spontaneity. I produce clear and detailed texts on various topics and can argue a viewpoint on general issues.	My English level is advanced. I understand a wide variety of extensive and complex texts, recognizing implicit meanings. I express myself fluently and spontaneously, using the language flexibly and effectively for social, academic, and professional purposes. I can produce clear, well-structured, and detailed texts on complex subjects.
Achieved Level					
Achievements and Areas for Improvement					

**Table:** 

Habilidad Transversal	Muy Bajo	Bajo	Medio	Medio Alto	Muy Alto
**Tecnología Digital y Gestión del Conocimiento** ¿En qué grado usas la tecnología para buscar y aplicar información en la solución de problemas reales?	Uso el celular o redes sociales para buscar información básica, sin verificar las fuentes.	Utilizo buscadores como Google y programas básicos (Word, PowerPoint) para organizar información, pero no analizo su calidad.	Uso bases de datos académicas y herramientas como Excel para buscar y analizar información relacionada con problemas de mi entorno.	Manejo software especializado para analizar información compleja y proponer soluciones a problemas reales en mi campo de estudio.	Utilizo bases de datos científicas para investigar y resolver problemas complejos. Ayudo a otros a usar la tecnología para gestionar información eficazmente.
Nivel obtenido					
Logros y aspectos por mejorar					
**Habilidades Socioemocionales** ¿En qué grado manejas tus habilidades sociales y emocionales para afrontar los problemas de la vida?	Me afectan mucho las situaciones difíciles y tengo conflictos frecuentes con otros.	Intento respetar a los demás, pero me cuesta controlar mis emociones en situaciones de estrés.	Controlo mis emociones para no afectar a otros y puedo entender diferentes puntos de vista en conflictos.	Manejo efectivamente mis emociones para lograr metas y mejorar la convivencia, usando técnicas como la relajación.	Uso mis habilidades emocionales para mejorar mi entorno, ayudando a otros a resolver conflictos y fomentando un ambiente positivo.
Nivel obtenido					
Logros y aspectos por mejorar					
**Trabajo Colaborativo** ¿En qué grado trabajas de manera colaborativa con otras personas para resolver los problemas del entorno?	Prefiero trabajar solo, evitando colaborar incluso cuando es necesario.	Participo en trabajos grupales haciendo lo mínimo requerido, sin tomar iniciativas.	Colaboro activamente en equipos, aportando ideas y cumpliendo mis responsabilidades para resolver los problemas.	Me esfuerzo por mejorar mi desempeño y el del equipo, proponiendo soluciones a los problemas que surgen.	Lidero equipos eficazmente, resolviendo conflictos y motivando a otros para lograr metas de alto impacto, como también para resolver problemas retadores.
Nivel obtenido					
Logros y aspectos por mejorar					
**Formación Continua** ¿En qué grado buscas formarte y capacitarte por tu propia iniciativa para afrontar los problemas del entorno?	Solo realizo las actividades de formación obligatorias en mis estudios.	Asisto ocasionalmente a actividades extra de formación, principalmente para mejorar mi currículum.	Busco activamente oportunidades de aprendizaje para prepararme mejor para los problemas del entorno.	Evalúo mis necesidades de formación y busco capacitación específica para mejorar mis habilidades.	Gestiono mi aprendizaje continuo como parte de un plan de vida, y ayudo a otros a mejorar sus habilidades.
Nivel obtenido					
Logros y aspectos por mejorar					
**Pensamiento Complejo** ¿En qué grado analizas y resuelves los problemas con pensamiento crítico, creatividad y flexibilidad?	Me cuesta adaptar mi forma de pensar y tiendo a seguir las opiniones de otros.	Puedo analizar problemas simples, pero me es difícil ver diferentes perspectivas.	Abordo los problemas con flexibilidad y soy capaz de cambiar de opinión en función de los hechos y los argumentos.	Evalúo críticamente información de diversas fuentes y ajusto mis estrategias según cada situación.	Articulo conocimientos de varias áreas para resolver problemas complejos de forma creativa e innovadora.
Nivel obtenido					
Logros y aspectos por mejorar					
**Liderazgo** ¿En qué grado influyes positivamente en otros para mejorar tu entorno?	Identifico metas básicas pero no tomo iniciativas de liderazgo.	Puedo planificar para lograr objetivos, pero me cuesta influir en otros.	Desarrollo planes para resolver problemas en mi comunidad y empiezo a guiar a otros.	Gestiono proyectos que impactan positivamente en mi entorno, promoviendo la inclusión.	Lidero cambios significativos, empoderando a otros para mejorar las condiciones de vida en la comunidad.
Nivel obtenido					
Logros y aspectos por mejorar					
**Investigación** ¿En qué grado participas en la creación de nuevo conocimiento a través de la investigación?	Conozco la idea básica de investigación, pero no he participado en proyectos.	He ayudado en tareas simples de investigación, como aplicar encuestas.	He participado en actividades de investigación con compañeros o profesores.	Participo activamente en proyectos de investigación y he presentado resultados en eventos académicos.	Contribuyo en la publicación de artículos científicos y lidero proyectos de investigación sobre problemas relevantes.
Nivel obtenido					
Logros y aspectos por mejorar					
**Comunicación** ¿En qué grado expresas tus ideas de forma clara y efectiva, de manera oral y por escrito?	Tengo dificultades para expresarme claramente, tanto hablando como escribiendo.	Puedo comunicar ideas simples, pero con limitaciones de vocabulario y estructura.	Expongo ideas claramente en presentaciones e informes sobre temas familiares.	Presento argumentos sólidos y coherentes al abordar problemas complejos.	Comunico ideas de manera persuasiva y ayudo a otros a mejorar sus habilidades de comunicación.
Nivel obtenido					
Logros y aspectos por mejorar					
**Emprendimiento** ¿En qué grado identificas oportunidades y las aprovechas para crear valor en tu entorno?	Solo realizo las actividades que me asignan, sin buscar nuevas oportunidades.	Tengo ideas para resolver problemas, pero aún no las he puesto en práctica.	He participado en iniciativas para resolver necesidades en mi entorno cercano.	Lidero proyectos que abordan problemas relevantes en mi comunidad.	Desarrollo proyectos innovadores con impacto significativo y fomento el emprendimiento en otros.
Nivel obtenido					
Logros y aspectos por mejorar					
**Inglés** ¿Cuál es tu nivel de inglés?	Conozco solo algunas palabras en inglés y puedo formar frases muy elementales. Mi comprensión y expresión son extremadamente limitadas.	Mi nivel de inglés es elemental. Puedo hacer y responder preguntas simples sobre situaciones cotidianas, pero con dificultad y errores frecuentes.	Mi nivel de inglés es básico-intermedio. Comprendo e interpreto expresiones y frases de uso frecuente relacionadas con áreas de experiencia relevantes (familia, trabajo, estudios). Puedo comunicarme en tareas sencillas y habituales que requieren un intercambio simple de información.	Mi nivel de inglés es intermedio-avanzado. Puedo relacionarme con hablantes nativos con un grado suficiente de fluidez y naturalidad. Produzco textos claros y detallados sobre diversos temas y puedo argumentar un punto de vista sobre temas generales.	Mi nivel de inglés es avanzado. Comprendo una amplia variedad de textos extensos y complejos, reconociendo sentidos implícitos. Me expreso con fluidez y espontaneidad, usando el idioma de manera flexible y eficaz para fines sociales, académicos y profesionales. Puedo producir textos claros, bien estructurados y detallados sobre temas complejos.
Nivel obtenido					
Logros y aspectos por mejorar					

**Table:** 

Indicator	Very Low	Low	Medium	Medium High	Very High
**Community issues** How extensively are community issues addressed during classes?	We only see theory, with no connection to real problems.	Community issues are occasionally mentioned but not analyzed.	We analyze community issues to better understand the topics.	We frequently work on real community problems and propose solutions.	We always develop projects to solve concrete community issues and follow up on solutions.
Achieved Level					
Achievements and Areas for Improvement					
**Inclusion** To what extent is it ensured that all students learn and participate in study activities, with no one left behind?	There is no support for students with difficulties.	Limited support is available and only for some students.	General support is available but not personalized.	Various forms of support and individual tracking are offered.	There is a comprehensive support system with various strategies to ensure that all students achieve their goals.
Achieved Level					
Achievements and Areas for Improvement					
**Student management** To what extent do students participate in organizing classroom activities, assisting teachers in their management?	The teacher organizes everything without our participation.	We participate minimally, only in simple tasks.	We have assigned roles but with little real responsibility.	We actively participate in organization and have significant responsibilities.	We manage a large part of the activities and support the teacher as co-organizers.
Achieved Level					
Achievements and Areas for Improvement					
**Interactive and dynamic activities** How dynamic and interactive are the classes, and how motivating and participatory?	Classes are monotonous, and we only listen to the teacher.	There is little interaction and few varied activities.	Classes combine explanations with some practical activities.	Classes are varied with many participative activities.	Classes are very dynamic, including games, projects, and innovative activities.
Achieved Level					
Achievements and Areas for Improvement					
**Collaborative environment** To what extent is there a trusting environment where teamwork is used to learn and solve problems?	We mostly work alone.	Sometimes we work in groups, but we do not assist each other much.	We work in teams but with undefined roles.	There is a trusting atmosphere in the group, and teamwork is organized.	There is great trust and effective collaboration to learn and solve problems.
Achieved Level					
Achievements and Areas for Improvement					
**Formative assessment** To what extent do assessments help students to continuously learn and improve?	We only receive grades without feedback.	Feedback is general and not very useful.	We receive comments that help us improve.	Continuous self-assessment is encouraged for learning and improvement.	We provide support for improvement and strengthen accountability and honesty in assessment.
Achieved Level					
Achievements and Areas for Improvement					
**Social commitment** To what extent do classes aim for students to have a commitment to society and help improve living conditions?	Social commitment is not addressed.	Social issues are discussed but without concrete actions.	We analyze social issues and propose solutions.	We develop projects with real social impact.	We implement solutions and actively commit to the community.
Achieved Level					
Achievements and Areas for Improvement					
**Critical analysis** To what extent is critical thinking applied in class to analyze information and study the environment?	We only memorize information without analyzing it.	We analyze information superficially.	Critical analysis is applied to some topics.	We frequently analyze issues critically.	We always use critical thinking to analyze problems and propose solutions.
Achieved Level					
Achievements and Areas for Improvement					
**Digital technology** To what extent are students taught to use technology to solve community problems?	We use very little technology in class. We only see simple presentations and PDF documents.	We use some basic digital tools, such as videos and online quizzes, but not for solving real problems.	We use basic digital tools such as videos, blogs, online quizzes, and internet communication systems for learning.	We use various digital applications for learning and solving community issues, with critical analysis.	We are trained in the use of digital tools to employ them with critical thinking and creativity.
Achieved Level					
Achievements and Areas for Improvement					

**Table:** 

Pregunta	Muy Bajo	Bajo	Medio	Medio Alto	Muy Alto
**Problemas de la comunidad** ¿En qué grado se abordan problemas de la comunidad durante las clases?	Solo vemos teoría, sin conexión con problemas reales.	Ocasionalmente se mencionan problemas de la comunidad, pero no los analizamos.	Analizamos problemas de la comunidad para entender mejor los temas.	Frecuentemente trabajamos en problemas reales de la comunidad y proponemos soluciones.	Siempre desarrollamos proyectos para resolver problemas concretos de la comunidad y damos seguimiento a las soluciones.
Nivel obtenido					
Logros y aspectos por mejorar					
**Inclusión** ¿En qué grado se busca que todos los estudiantes aprendan y participen en las actividades de estudio, y nadie se quede atrás?	No hay apoyo para estudiantes con dificultades.	Existe poco apoyo y solo para algunos estudiantes.	Hay apoyo general pero no es personalizado.	Se ofrecen diferentes formas de apoyo y seguimiento individual.	Existe un sistema completo de apoyo con estrategias diversas para que todos los estudiantes alcancen los resultados de aprendizaje.
Nivel obtenido					
Logros y aspectos por mejorar					
**Gestión de los estudiantes** ¿En qué grado se busca que los estudiantes participen en la organización de las actividades de clase, ayudando a los docentes en su gestión?	El profesor organiza todo sin nuestra participación.	Participamos muy poco, solo en tareas simples.	Tenemos roles asignados, pero con poca responsabilidad real.	Participamos activamente en la organización y tenemos responsabilidades importantes.	Gestionamos gran parte de las actividades y apoyamos al profesor como co-organizadores.
Nivel obtenido					
Logros y aspectos por mejorar					
**Actividades interactivas y dinámicas** ¿En qué grado las clases son dinámicas e interactivas, motivantes y participativas?	Las clases son monótonas y solo escuchamos al profesor.	Hay poca interacción y pocas actividades diferentes.	Las clases combinan explicaciones con algunas actividades prácticas.	Las clases son variadas con muchas actividades participativas.	Las clases son muy dinámicas con juegos, proyectos y actividades innovadoras.
Nivel obtenido					
Logros y aspectos por mejorar					
**Ambiente colaborativo** ¿En qué grado se tiene un ambiente de confianza y se trabaja en equipo para aprender y resolver los problemas?	Casi siempre trabajamos solos.	A veces trabajamos en grupo, pero no nos ayudamos mucho.	Trabajamos en equipo, pero con roles poco definidos.	Hay un ambiente de confianza en el grupo y trabajo en equipo organizado.	Existe gran confianza y colaboración efectiva para aprender y resolver problemas.
Nivel obtenido					
Logros y aspectos por mejorar					
**Evaluación formativa** ¿En qué grado las evaluaciones ayudan a los estudiantes a aprender, pensar de manera crítica y mejorar de manera continua?	Solo recibimos calificaciones sin retroalimentación.	La retroalimentación es general y poco útil.	Recibimos comentarios que nos ayudan a mejorar.	Se promueve la autoevaluación continua para aprender y mejorar	Tenemos apoyo para mejorar y fortalecemos la responsabilidad y la honestidad en la evaluación
Nivel obtenido					
Logros y aspectos por mejorar					
**Compromiso social** ¿En qué grado se busca en las clases que los estudiantes tengan compromiso con la sociedad y contribuyan a mejorar las condiciones de vida?	No se aborda el compromiso social.	Se habla de problemas sociales pero sin acciones concretas.	Analizamos problemas sociales y propuestas de solución.	Desarrollamos proyectos con impacto social real.	Implementamos soluciones y nos comprometemos activamente con la comunidad.
Nivel obtenido					
Logros y aspectos por mejorar					
**Análisis crítico** ¿En qué grado se aplica en las clases el pensamiento crítico en el análisis de la información y el estudio del entorno?	Solo memorizamos información sin analizarla.	Analizamos información de manera superficial.	Aplicamos análisis crítico en algunos temas.	Frecuentemente analizamos problemas de manera crítica.	Siempre usamos pensamiento crítico para analizar problemas y proponer soluciones.
Nivel obtenido					
Logros y aspectos por mejorar					
**Tecnología digital** ¿En qué grado se aplica en las clases la tecnología digital para resolver problemas de la comunidad y aprender?	Usamos muy poca tecnología en clase. Solo vemos presentaciones simples y documentos PDF.	Utilizamos algunas herramientas digitales básicas, como videos y cuestionarios en línea, pero no para resolver problemas reales.	Usamos herramientas digitales básicas como videos, blogs, cuestionarios en línea o sistemas de comunicación por internet para aprender.	Usamos diversas aplicaciones digitales para aprender y resolver problemas de la comunidad, con análisis crítico.	Nos formamos en el uso de las herramientas digitales para emplearlas con pensamiento crítico y creatividad.
Nivel obtenido					
Logros y aspectos por mejorar					

**Table:** 

Socioformative Learning Strategy	Very Low Level	Low Level	Medium Level	High Level	Very High Level
**1. Class Preparation** How do you prepare before classes?	I don't review materials or engage in any preparatory activities before classes.	I superficially review some topics before classes without deepening my understanding.	I sometimes dedicate time before each class to review topics and complete basic exercises, identifying main concepts.	I regularly review materials and complete exercises before classes.	I thoroughly review materials before classes, read them, analyze examples, and complete comprehension exercises such as graphic organizers.
Achieved Level					
Achievements and Areas for Improvement					
**2. Class Participation** What is your level of participation in classes?	I attend class but don't take notes or ask questions, even when I don't understand the topics.	I take intermittent verbatim notes and ask 1–2 basic questions per class when the professor requests participation.	I take notes in my own words, formulate 2–3 questions per class to clarify doubts, and participate when the professor asks.	I create graphic organizers during class, formulate 3–4 questions linking concepts, and voluntarily participate by providing examples.	I develop comprehensive concept maps, formulate 4–5 analytical questions, propose practical examples, and generate discussions that enrich the class.
Achieved Level					
Achievements and Areas for Improvement					
**3. Information Search** How do you search for and use relevant information in your studies?	I only use the material provided in class, without seeking additional information.	I search for information on general websites like Wikipedia, without verifying source reliability.	I use Google Scholar and at least one specialized database, organizing information in digital folders.	I consult multiple academic databases, verify source currency, and create summary cards with main ideas.	I access specialized databases, analyze recent scientific articles, compare authors, and create personal databases with relevant information.
Achieved Level					
Achievements and Areas for Improvement					
**4. Problem Solving** How do you apply strategies to solve academic and environmental problems?	I attempt to solve problems without a specific method, seeking quick completion without verifying results.	I follow textbook examples or professor explanations without adapting them to the specific problem.	I identify key problem elements, propose at least two possible solutions, and verify the most viable one.	I develop a structured solution plan, monitor each process step, and evaluate results using specific criteria.	I analyze problems from multiple perspectives, propose innovative solutions based on scientific evidence, and verify their effectiveness using rigorous methods.
Achieved Level					
Achievements and Areas for Improvement					
**5. Collaborative Work** How do you work with others to achieve common goals?	I avoid participating in group work and, when I do, I don't contribute to the team.	I attend team meetings but only complete assigned tasks without proposing ideas.	I actively participate in the team, fulfill my responsibilities, and contribute at least two ideas per session.	I propose innovative ideas, support my peers when they face difficulties, and coordinate at least one group activity.	I lead group projects, facilitate conflict resolution, promote everyone's participation, and establish challenging team goals.
Achieved Level					
Achievements and Areas for Improvement					
**6. Self-Assessment and Improvement** How do you self-assess to continually improve?	I don't engage in any reflection about my academic performance.	I review my grades but don't analyze the causes of my mistakes or implement changes.	I assess my achievements and challenges in the learning process, set goals, but find it difficult to improve.	I critically evaluate my learning performance and make some improvements.	I critically and humbly evaluate my learning performance, plan actions for improvement, and continually enhance my skills.
Achieved Level					
Achievements and Areas for Improvement					
**7. Digital Technology** How do you use technology to manage your learning and projects?	I don't use digital technologies in my learning process, only using my phone for social media and entertainment.	I use basic technologies like general search engines and office applications (Word, PowerPoint) for simple tasks.	I employ educational digital technologies such as academic databases, organization applications, and online collaboration tools to enhance my learning.	I use advanced digital tools to analyze complex information, create multimedia content, and manage collaborative projects. I ethically leverage emerging technologies to enhance my learning.	I expertly integrate multiple digital technologies into my learning process, develop my own resources, advise others on effective digital tool use, and stay updated on new educational technologies.
Achieved Level					
Achievements and Areas for Improvement					
**8. Academic Communication** How do you communicate in written and oral forms?	I cannot express my ideas clearly either orally or in writing.	I communicate basic ideas simply, with limited vocabulary and unclear structure.	I express ideas clearly, use basic academic vocabulary, and maintain a logical structure in my presentations.	I effectively communicate complex ideas, adapt my discourse to context, and use multimodal resources to enhance understanding.	I develop high-impact presentations, argue with scientific evidence, adapt my communication to diverse audiences, and advise others on their communication skills.
Achieved Level					
Achievements and Areas for Improvement					
**9. Projects with Social Impact** Do you participate in projects to develop your skills?	I don't connect my learning with real community problems.	I identify problems in my environment but don't propose concrete solutions.	I develop at least one project that addresses a local need, documenting the process and basic outcomes.	I lead two semester projects that positively impact my community, measuring results and generating evidence of improvement.	I manage a network of social projects linked to my learning, involve other students and organizations, and publish results to inspire new initiatives.
Achieved Level					
Achievements and Areas for Improvement					

**Table:** 

Estrategia de aprendizaje socioformativa	Nivel Muy Bajo	Nivel Bajo	Nivel Medio	Nivel Medio Alto	Nivel Muy Alto
**1. Preparación antes de las clases** ¿Cómo te preparas para actividades de aprendizaje?	No reviso los materiales ni realizo ninguna actividad de preparación antes de las clases.	Reviso superficialmente algunos temas antes de las clases, sin profundizar en su comprensión.	A veces dedico tiempo antes de cada clase a revisar los temas y realizar ejercicios básicos, identificando conceptos principales.	Regularmente reviso los materiales y hago ejercicios antes de las clases	Reviso con profundidad los materiales antes de las clases, los leo, analizo ejemplos y hago ejercicios de comprensión, como organizadores gráficos.
Nivel obtenido					
Logros y aspectos por mejorar					
**2. Participación en clase** ¿Cuál es tu nivel de participación en las clases presenciales o virtuales?	Asisto a clase pero no tomo notas ni formulo preguntas, incluso cuando no comprendo los temas.	Tomo notas textuales de manera intermitente y hago 1–2 preguntas básicas por clase cuando el profesor lo solicita.	Tomo notas con mis propias palabras, formulo 2–3 preguntas por clase para aclarar dudas y participo cuando el profesor lo pide.	Elaboro organizadores gráficos durante la clase, formulo 3–4 preguntas relacionando conceptos y participo voluntariamente aportando ejemplos.	Desarrollo mapas conceptuales completos, formulo 4–5 preguntas de análisis, propongo ejemplos prácticos y genero discusiones que enriquecen la clase.
Nivel obtenido					
Logros y aspectos por mejorar					
**3. Búsqueda de información** ¿Cómo buscas y utilizas información pertinente en el estudio?	Utilizo únicamente el material proporcionado en clase, sin buscar información adicional.	Busco información en sitios web generales como Wikipedia, sin verificar la confiabilidad de las fuentes.	Utilizo Google Académico y al menos una base de datos especializada, organizando la información en carpetas digitales.	Consulto múltiples bases de datos académicas, verifico la actualidad de las fuentes y elaboro fichas de resumen con ideas principales.	Accedo a bases de datos especializadas, analizo artículos científicos recientes, contrasto autores y creo bases de datos personales con la información relevante.
Nivel obtenido					
Logros y aspectos por mejorar					
**4. Resolución de problemas** ¿Cómo aplicas estrategias para resolver problemas académicos y del entorno?	Intento resolver los problemas sin un método específico, buscando terminar rápidamente sin verificar resultados.	Sigo los ejemplos del libro o las explicaciones del profesor sin adaptarlos al problema específico.	Identifico los elementos clave del problema, propongo al menos dos soluciones posibles y verifico la más viable.	Desarrollo un plan estructurado de solución, monitoreo cada paso del proceso y evalúo los resultados con criterios específicos.	Analizo el problema desde múltiples perspectivas, propongo soluciones innovadoras basadas en evidencia científica y verifico su efectividad con métodos rigurosos.
Nivel obtenido					
Logros y aspectos por mejorar					
**5. Trabajo colaborativo** ¿Cómo trabajas con otros para lograr metas comunes y aprender?	Evito participar en trabajos grupales y, cuando lo hago, no aporto al equipo.	Asisto a las reuniones de equipo pero solo realizo las tareas que me asignan, sin proponer ideas.	Participo activamente en el equipo, cumplo mis responsabilidades y contribuyo con al menos dos ideas por sesión.	Propongo ideas innovadoras, apoyo a mis compañeros cuando tienen dificultades y coordino al menos una actividad grupal.	Lidero proyectos grupales, facilito la resolución de conflictos, promuevo la participación de todos y establezco metas desafiantes para el equipo.
Nivel obtenido					
Logros y aspectos por mejorar					
**6. Autoevaluación y mejora** ¿Cómo te autoevalúas para mejorar continuamente?	No realizo ningún tipo de reflexión sobre mi desempeño académico.	Reviso mis calificaciones, pero no analizo las causas de mis errores ni implemento cambios.	Evalúo mis logros y dificultades en el proceso de aprendizaje, establezco propósitos, pero se me dificulta mejorar.	Evalúo mi desempeño en el aprendizaje de manera crítica y hago algunas mejoras.	Evalúo mi desempeño en el aprendizaje con pensamiento crítico y humildad, planifico acciones de mejora y mejoro continuamente.
Nivel obtenido					
Logros y aspectos por mejorar					
**7. Tecnología digital** ¿Cómo empleas la tecnología para gestionar tu aprendizaje y proyectos?	No empleo tecnologías digitales en mi proceso de aprendizaje, solo uso el celular para redes sociales y entretenimiento.	Uso tecnologías básicas como buscadores generales y aplicaciones de oficina (Word, PowerPoint) para realizar tareas simples.	Empleo tecnologías digitales educativas como bases de datos académicas, aplicaciones de organización y herramientas de colaboración en línea para mejorar mi aprendizaje.	Utilizo herramientas digitales avanzadas para analizar información compleja, crear contenido multimedia y gestionar proyectos colaborativos. Aprovecho las tecnologías emergentes de manera ética para potenciar mi aprendizaje.	Integro de manera experta múltiples tecnologías digitales en mi proceso de aprendizaje, desarrollo recursos propios, asesoro a otros en el uso efectivo de herramientas digitales y me mantengo actualizado sobre nuevas tecnologías educativas.
Nivel obtenido					
Logros y aspectos por mejorar					
**8. Comunicación académica** ¿Cómo te comunicas a nivel escrito y oral?	No logro expresar mis ideas claramente ni de forma oral ni escrita.	Comunico ideas básicas de manera simple, con vocabulario limitado y estructura poco clara.	Expreso ideas con claridad, utilizo vocabulario académico básico y mantengo una estructura lógica en mis presentaciones.	Comunico ideas complejas eficazmente, adapto mi discurso según el contexto y utilizo recursos multimodales para mejorar la comprensión.	Desarrollo presentaciones de alto impacto, argumento con evidencia científica, adapto mi comunicación a diversos públicos y asesoro a otros en sus habilidades comunicativas.
Nivel obtenido					
Logros y aspectos por mejorar					
**9. Proyectos con impacto social** ¿Participas en proyectos para desarrollar tus habilidades?	No relaciono mi aprendizaje con problemas reales de la comunidad.	Identifico problemas en mi entorno, pero no propongo acciones concretas de solución.	Desarrollo al menos un proyecto que aborda una necesidad local, documentando el proceso y los resultados básicos.	Lidero algún proyecto que impacta positivamente a mi comunidad, midiendo resultados y generando evidencias de mejora.	Gestiono una red de proyectos sociales vinculados a mi aprendizaje, involucro a otros estudiantes y organizaciones, y publico los resultados para inspirar nuevas iniciativas.
Nivel obtenido					
Logros y aspectos por mejorar					

**Table:** 

Aspecto	Descripción	Nivel Muy Bajo	Nivel Bajo	Nivel Medio	Nivel Medio Alto	Nivel Muy Alto
1. Satisfacción con el Estudio en Línea.	Valoración integral de la experiencia de aprendizaje en el entorno virtual como complemento de la educación presencial, considerando aspectos como la calidad de los contenidos, la efectividad de las estrategias de enseñanza y el cumplimiento de sus expectativas de aprendizaje.					
2. Grado de Utilidad de las Actividades en Línea	Es el grado de percepción sobre el valor práctico y la aplicabilidad de las actividades realizadas en el entorno virtual, considerando su relevancia para el desarrollo de competencias, la resolución de problemas del contexto y la contribución a su formación integral.					
3. Amigabilidad del Aula Virtual	Facilidad de uso, navegación e interacción con la plataforma de aprendizaje en línea, incluyendo la intuitividad de la interfaz, la accesibilidad de los recursos y la claridad en la organización de los elementos.					
4. Organización del Aula Virtual	Se refiere a la estructura lógica, orden y coherencia de los contenidos, actividades y recursos en el entorno virtual, incluyendo la claridad en las instrucciones, la secuenciación del aprendizaje y la disposición de los elementos formativos.					
5. Interacción en el Aula Virtual	Se refiere a la facilidad para interactuar con los contenidos y pares, para hacer comentarios, tener retroalimentación,compartir, comunicarse con los pares, hacer menciones de estos, establecer contactos, etc.					
6. Grado de Atracción	Es el grado de motivación que genera el aula virtual con sus contenidos y actividades para que los estudiantes deseen ingresar y permanecer en ella. Esto implica tener recursos y materiales que se adecúen a los intereses de los educandos.					
7. Adaptavilidad del Aula	Es la capacidad del entorno virtual para ajustarse a las necesidades, preferencias y ritmos de aprendizaje individuales de los estudiantes, incluyendo la personalización de contenidos y actividades según el desempeño e intereses particulares.					
Logros						
Aspectos para mejorar						

**Table:** 

Element	Description	Very Low Level	Low Level	Medium Level	High Level	Very High Level
Satisfaction with Online Learning	Comprehensive assessment of the learning experience in a virtual environment as a supplement to face-to-face education, considering quality of content, effectiveness of teaching strategies, and fulfillment of learning expectations.					
Utility of Online Activities	Degree of perception about the practical value and applicability of activities in the virtual environment, relevant to competency development, problem-solving, and holistic education.					
User-friendliness of the Virtual Classroom	Ease of use, navigation, and interaction with the online learning platform, including intuitiveness of the interface, accessibility of resources, and clarity in the organization of elements.					
Organization of the Virtual Classroom	Refers to the logical structure, order, and coherence of content, activities, and resources in the virtual environment, including clarity of instructions, sequencing of learning, and arrangement of educational elements.					
Interaction in the Virtual Classroom	Refers to the ease of interacting with content and peers, enabling comments, feedback, sharing, communication with peers, mentions, and establishing contacts.					
Degree of Engagement	Level of motivation generated by the virtual classroom through its content and activities that encourage students to log in and stay engaged, tailored to the interests of the learners.					
Adaptability of the Classroom	Capability of the virtual environment to adapt to the individual needs, preferences, and learning paces of students, including personalization of content and activities based on performance and interests.					
Achieved Level						
Areas for Improvement						

## Data Availability

The Open Science Framework (OSF) repository (https://doi.org/10.17605/OSF.IO/WQEM8) includes the instruments in Word format for download in both Spanish and English, as well as a report on the design process of each of the three rubrics used in this study. This report is based on the recommendations and reporting instrument of Ernesto Panadero, Maryam Alqassab, Javier Fernández Ruiz, and Jose Carlos G. Ocampo (https://doi.org/10.17605/OSF.IO/5K42Z). The instruments may be utilized by anyone without the need for permission. The sole requirement is to cite this article and the original document by Dr. Tobón (2020).

## **Note: The 4 instruments presented below in both English and Spanish should be cited as follows:**

Guerra-Macías, Y., & Tobón, S. (2024). Development of transversal skills in higher education programs in conjunction with online learning: relationship between learning strategies, project-based pedagogical practices, e-learning platforms, and academic performance. Heliyon. https://doi.org/10.1016/j.heliyon.2024.e41099.

## Appendix A Transversal Skills Rubric

### Presentation

This rubric is designed to assess the level of development in ten cross-curricular skills, also known as 21st-century skills, soft skills, or key competencies. These skills are recognized as essential for professional development and for contributing to social sustainability in Latin America, although they can be adapted for use in other regions with the necessary cultural and linguistic adjustments. The skills evaluated are defined and described based on the socioformative pedagogical model, which links education to the needs and challenges of society. Consequently, they are approached from a perspective that emphasizes community problem-solving, critical analysis of the environment, ethics, and creative thinking.

### Rubric Objectives

• **Assess the Development Level:** Analyze your proficiency level in each of the ten cross-functional skills outlined in the rubric below, which are essential for addressing the challenges of today's society.
• **Identify Strengths and Areas for Improvement:** Use critical self-assessment to pinpoint your achievements and areas where you can enhance performance in each of the ten skills.

#### Intended Audience

This rubric is designed for a broad audience, including:

• Students in secondary, high school, and higher education
• Educators
• Professionals
• Individuals with at least a high school or equivalent education

#### Instructions

To use this rubric effectively, follow these instructions:

1. **Review the Rubric:** Familiarize yourself with the rubric's structure before starting your self-assessment. Understanding the skills and the criteria for each level will facilitate a more effective evaluation.
2. **Respond in Sequence:** It's best to answer starting from question 1 and continue in order. However, if a particular question inspires you, feel free to start there and then address the remaining questions.
3. **Interpret Each Question Carefully:** Take the time to thoroughly understand the intent and meaning of each question before responding.
4. **Understand Performance Levels:** Below is a summary of each performance level:
  • **Very Low Level:** You are beginning to develop the skill.
  • **Low Level:** You have basic knowledge but need further practice.
  • **Medium Level:** You handle the fundamental aspects of the skill.
  • **Medium-High Level:** You demonstrate solid proficiency and can make independent judgments.
  • **Very High Level:** You have mastered the skill and can instruct others.
5. **Start from the Lowest Level:** Begin your assessment at the lowest level and work your way up. Mark an “X" at the level that accurately reflects your current ability for each skill. Be honest; if you only partially meet the criteria for a level, consider the next lower level as your true current level.
6. **Document Achievements and Areas for Improvement:** Under each question, note specific accomplishments and identify areas that need development, guided by the descriptions of each level. For instance, if you assess yourself as “Medium” in “Collaborative Work,” describe a specific situation where you contributed ideas and fulfilled responsibilities within a team, then outline what would be needed to reach “Medium-High” or “Very High” levels.

Best of luck with your self-assessment!

## Appendix B Rúbrica de Habilidades Transversales

(Versión en español para Latinoamérica)

### Presentación

Esta rúbrica está diseñada para ayudar a determinar el nivel de desarrollo de diez habilidades transversales, también conocidas como habilidades del siglo XXI, capacidades blandas o competencias clave. Estas habilidades se han identificado como esenciales para el desarrollo profesional y para contribuir a la sostenibilidad social en América Latina, aunque pueden ser aplicables en otras regiones con las adaptaciones culturales y lingüísticas pertinentes. Las habilidades evaluadas se definen y describen a partir del modelo pedagógico de la socioformación, que vincula la educación con las necesidades y retos de la sociedad. Por lo tanto, se abordan desde una perspectiva que enfatiza en la problematización de la comunidad, el análisis crítico del entorno, la ética y el pensamiento creativo.

### Propósitos de la rúbrica

**Evaluar el Nivel de Desarrollo:** Analiza qué nivel de desarrollo posees en cada una de las diez habilidades transversales que se presentan en la siguiente rúbrica, las cuales son esenciales para afrontar los retos de la sociedad actual.

**Identificar Fortalezas y Áreas de Mejora:** Utiliza la autoevaluación crítica para determinar tus logros y las áreas donde puedes mejorar el desempeño en cada una de las diez habilidades.

#### Dirigida a:

Esta rúbrica está diseñada para una amplia audiencia, incluyendo:

- Estudiantes de secundaria, educación media y educación superior
- Docentes
- Profesionales
- Empleados con formación mínima de secundaria o educación media

#### Instrucciones:

Emplea la presente rúbrica considerando las siguientes instrucciones:

1. **Revisa la rúbrica antes de empezar tu autoevaluació**n. Esto es importante para que comprendas la estructura y así te orientes mejor en el proceso. Necesitas saber qué habilidades vas a evaluar en ti y qué significa cada nivel.
2. **Responde en orden.** Sugerimos iniciar la autoevaluación en la pregunta 1, e ir avanzando en orden. Sin embargo, puedes empezar también por la pregunta que más te motive y luego completar lo demás.
3. **Comprende cada pregunta antes de responder.** Revisa cada pregunta de manera crítica y lo que significa, antes de comenzar a responder.
4. **Comprende los niveles antes de autoevaluarte.** A continuación, se presenta una síntesis general de cada nivel de desempeño:
  • Nivel muy bajo: Apenas estás empezando a desarrollar la habilidad
  • Nivel bajo: Tienes nociones básicas de acercamiento a la habilidad, pero necesitas práctica
  • Nivel medio: Manejas los elementos básicos de la habilidad
  • Nivel medio alto: Tienes buen dominio y criterio propio
  • Nivel muy alto: Dominas la habilidad y puedes enseñar a otros
5. **Inicia desde el nivel más bajo.** Para cada habilidad, comienza evaluándote desde el nivel muy bajo y luego ve avanzando hacia arriba, en orden. Marca con una “X" el nivel que mejor describe tu situación actual, debajo de cada pregunta. Es importante responder con honestidad. Si cumples solo una parte de un nivel, pero no todo, tu nivel real es el anterior que sí cumples por completo.
6. **Logros y aspectos por mejorar.** Debajo de cada pregunta, en el espacio destinado para ello, determina los logros concretos y los aspectos específicos que debes mejorar en cada habilidad, guiándote por los descriptores de los niveles. Por ejemplo, si en “Trabajo Colaborativo” marcas “Medio”, escribe una situación específica donde contribuiste con ideas y cumpliste tus responsabilidades en un equipo. Luego, señala los aspectos a mejorar tomando en cuenta lo que implican los niveles medio alto y muy alto.

Muchos éxitos en tu autoevaluación.

## Appendix C Teaching Based on Socioformative Projects Rubric (students)

### Presentation

This rubric is designed to assess the level of implementation of socioformative projects within the pedagogical practices of educational institutions or universities. Socioformative projects are a key component of the socioformative pedagogical model, involving student participation in the analysis and resolution of community problems to achieve specific learning outcomes. This approach emphasizes critical engagement with reality, collaborative work, ethical commitment, inclusion, enhancement of socioemotional skills, and the development of critical and creative thinking. The methodology can be applied within a specific course, across various subjects or modules, or through interdisciplinary and transdisciplinary processes within the framework of integrative projects.

This rubric is primarily aimed at students, to assess the degree of implementation of this methodology in their classes. Unlike other project-based methodologies such as the STEAM approach or traditional project-based learning, this rubric uniquely focuses on the critical and innovative handling of community issues, student inclusion in class management, formative assessment that promotes critical thinking, and social engagement. These elements are addressed flexibly without adhering to a strict sequence, allowing for a dynamic and reflective learning experience. By using this rubric, students and educators can identify achievements and areas for improvement in the educational strategies employed, thereby facilitating collective actions for enhancement in connection with the social environment.

### Purposes of the Rubric

• **Evaluate the Implementation Level**: Analyze in your classes the extent to which each of the 9 actions of socioformative projects presented in the rubric are addressed.
• **Identify Strengths and Areas for Improvement**: Critically evaluate the achievements and areas needing improvement in each of the 9 actions involved in working with socioformative projects in the classroom.

#### Intended Audience

• High school and middle school students
• College and university students

#### Instructions

To effectively utilize this rubric, follow these instructions:

1. **Review the Rubric**: Acquaint yourself with the rubric's structure before beginning the evaluation.
2. **Respond in Sequence**: It is best to respond starting from question 1 and proceeding in order. However, if a particular question inspires you, feel free to start there and then address the remaining questions.
3. **Interpret Each Question Carefully**: Take the time to fully understand the intent and meaning of each question before responding to the evaluation.
4. **Understand the Levels**: For each question, select the level at which each action of the projects applies in the classroom, considering its meaning:
  • **Very Low Level**: Virtually no implementation of the indicated action.
  • **Low Level**: Occasional and superficial implementation of the action or component.
  • **Medium Level**: Regular execution of the action or component, but without depth.
  • **Medium-High Level**: Consistent and relevant implementation of the action or component.
  • **Very High Level**: Continuous, profound, and highly relevant application of the action or component.
5. **Start from the Lowest Level**: Begin your evaluation at the lowest level and gradually move upward. Mark with an “X" the level that accurately reflects the degree to which the socioformative projects are currently applied. Respond honestly; if only partially meeting the criteria for a level, consider the next lowest level as the appropriate choice.
6. **Describe Achievements and Areas for Improvement**: Below each question, note specific achievements and potential areas for improvement in the implementation of each action of the socioformative projects.
  Good luck with your class evaluation!

## Appendix D Rúbrica de Docencia Basada en Proyectos Socioformativos (estudiantes)

(Versión en español para Latinoamérica)

### Presentación

Esta rúbrica está diseñada para evaluar el nivel de implementación de los proyectos socioformativos dentro de las prácticas pedagógicas en instituciones educativas o universidades. Los proyectos socioformativos son un componente clave del modelo pedagógico socioformativo, e implican la participación de los estudiantes en el análisis y la resolución de problemas comunitarios para alcanzar resultados de aprendizaje específicos. Este enfoque enfatiza en la problematización crítica de la realidad, el trabajo colaborativo, el compromiso ético, la inclusión, el fortalecimiento de habilidades socioemocionales y el desarrollo del pensamiento crítico y creativo. La metodología puede aplicarse dentro de un curso específico, a través de varias asignaturas o módulos, o mediante procesos interdisciplinarios y transdisciplinarios dentro del marco de proyectos integradores.

Esta rúbrica está orientada principalmente a los estudiantes, para evaluar el grado de implementación de esta metodología en sus clases. A diferencia de otras metodologías basadas en proyectos, como el enfoque STEAM o el enfoque tradicional del aprendizaje basado en proyectos, esta rúbrica se centra de manera única en el abordaje crítico e innovador de problemas comunitarios, la inclusión de los estudiantes en la gestión de las clases, la evaluación formativa que fomenta el pensamiento crítico y el compromiso social. Estos elementos se abordan de manera flexible sin seguir una secuencia estricta, permitiendo una experiencia de aprendizaje dinámica y reflexiva. Mediante el uso de esta rúbrica, los estudiantes y educadores pueden identificar los logros y los aspectos a mejorar en las estrategias educativas empleadas en el marco de la vinculación con el entorno social, para implementar acciones de mejora entre todos.

### Propósitos de la rúbrica

**Evaluar el Nivel de Implementación:** Analiza en tus clases el nivel en el cual se aborda cada una de las 9 acciones de los proyectos socioformativos que se presentan en la rúbrica.

**Identificar Fortalezas y Áreas de Mejora:** Evalúa de manera crítica los logros y los aspectos por mejorar en cada una de las 9 acciones que implica el trabajo con los proyectos socioformativos en las clases.

#### Dirigida a:

Esta rúbrica está diseñada para:

- Estudiantes de secundaria y educación media
- Estudiantes de educación superior

#### Instrucciones:

Para utilizar esta rúbrica de manera efectiva, sigue estas instrucciones:

1. **Revisar la Rúbrica:** Familiarízate con la estructura de la rúbrica antes de comenzar la evaluación.
2. **Responder en Secuencia:** Es mejor responder comenzando desde la pregunta 1 y continuar en orden. Sin embargo, si una pregunta en particular te inspira, siéntete libre de comenzar por ahí y luego aborda las preguntas restantes.
3. **Interpretar Cada Pregunta Cuidadosamente:** Tómate el tiempo para entender completamente la intención y el significado de cada pregunta antes de responder la evaluación.
4. **Entender los Niveles:** En cada pregunta, selecciona el nivel en el cual se aplica cada acción de los proyectos en las clases, considerando su significado:
  • Nivel Muy Bajo: Prácticamente no hay implementación de la acción indicada.
  • Nivel Bajo: Implementación ocasional y superficial de la acción o componente.
  • Nivel Medio: Ejecución regular de la acción o componente, pero sin profundidad.
  • Nivel Medio-Alto: Implementación constante y pertinente de la acción o componente.
  • Nivel Muy Alto: Aplicación continua, profunda y altamente relevante de la acción o componente.
5. **Comenzar desde el Nivel Más Bajo:** Comienza tu evaluación en el nivel más bajo y avanza hacia arriba, de manera gradual. Marca con una “X" el nivel que refleje con precisión el grado en el cual se aplican los proyectos socioformativos en este memento. Responde con honestidad; si solo se cumple parcialmente con los criterios de un nivel, considera el siguiente nivel más bajo como el nivel a elegir.
6. **Describir Logros y Aspectos para Mejorar:** Debajo de cada pregunta anota logros específicos y aspectos que se podrían mejorar en la implementación de cada acción de los proyectos socioformativos.

¡Buena suerte con la evaluación de tus clases!

## Appendix E Socioformative Rubric for Learning Strategies

### Presentation

This rubric has been designed to assess the extent to which students apply various strategies to achieve relevant and pertinent learning. Learning strategies are explicit or implicit procedures aimed at enhancing student learning by making it meaningful and applicable to real-world challenges. From the perspective of the socioformation pedagogical model, there is an emphasis on applying these strategies based on critical analysis and proposing solutions to community problems, creating collaborative and trust-based learning environments, and implementing projects with social and environmental impacts. Thus, these strategies are not limited to academic learning; they are comprehensive and systemic, considering holistic education, the management of contextual challenges, and the creation of stimulating learning environments or scenarios that foster personal development in interaction with others.

### Purposes of the Rubric

• Assess the Level of Implementation: Analyze the degree to which you implement a series of strategies to achieve the highest level of learning.
• Identify Strengths and Areas for Improvement: Critically evaluate the achievements and areas needing improvement for each of the 9 learning strategies presented, based on your experience.

#### Target Audience:

This rubric is intended for a broad audience, including:

• High school and secondary education students
• Higher education students

#### Instructions:

To use this rubric effectively, follow these instructions:

1. Review the Rubric: Familiarize yourself with the rubric's structure before starting the evaluation.
2. Respond in Sequence: It is best to answer starting from question 1 and proceed in order. However, if a particular question inspires you, feel free to begin there and then address the remaining questions.
3. Interpret Each Question Carefully: Take the time to fully understand the intent and meaning of each question before responding to the assessment.
4. Understand the Levels: For each question, select the level at which you apply the indicated learning strategy, considering the general meaning of the levels:
  • Very Low Level: Virtually no implementation of the learning strategy.
  • Low Level: Occasional and superficial use of the learning strategy.
  • Medium Level: Regular execution of the learning strategy, but without depth.
  • Medium-High Level: Consistent and relevant implementation of the learning strategy.
  • Very High Level: Continuous, in-depth, and highly relevant application of the learning strategy.
5. Start from the Lowest Level: Begin your evaluation at the lowest level and gradually work your way up. Mark with an “X" the level that accurately reflects the degree to which you apply each learning strategy. Respond honestly; if only partially meeting the criteria for a level, consider the next lower level as the appropriate choice.
6. Describe Achievements and Areas for Improvement: Beneath each question, note specific achievements and areas that could be improved in the application of each learning strategy.
  Good luck with your evaluation of learning strategies!

## Appendix F Rúbrica Socioformativa de Estrategias de Aprendizaje

(Versión en español para Latinoamérica)

### Presentación

Esta rúbrica ha sido diseñada para evaluar en qué medida los estudiantes aplican diversas estrategias para lograr un aprendizaje relevante y pertinente. Las estrategias de aprendizaje son procedimientos explícitos o implícitos que se orientan a mejorar el aprendizaje de los estudiantes, haciéndolo significativo y aplicable a los desafíos del mundo real. Desde la perspectiva del modelo pedagógico de la socioformación, se enfatiza en la aplicación de estas estrategias con base en el análisis crítico y la propuesta de soluciones a problemas comunitarios, la creación de ambientes de aprendizaje colaborativos y basados en la confianza, así como la implementación de proyectos con impacto social y ambiental. De este modo, estas estrategias no se limitan solo al aprendizaje académico; son integrales y sistémicas, considerando la formación integral, el manejo de retos contextuales y la creación de ambientes o escenarios de aprendizaje estimulantes del desarrollo personal en interacción con otros.

### Propósitos de la rúbrica

• **Evaluar el Nivel de Implementación:** Analiza en qué grado implementas una serie de estrategias para lograr el mayor grado de aprendizaje.
• **Identificar Fortalezas y Áreas de Mejora:** Evalúa de manera crítica los logros y los aspectos por mejorar en cada una de las 9 estrategias de aprendizaje que se presentan, con base en tu experiencia.

#### Dirigida a:

Esta rúbrica está diseñada para una amplia audiencia, incluyendo:

- Estudiantes de secundaria y educación media
- Estudiantes de educación superior

#### Instrucciones:

Para utilizar esta rúbrica de manera efectiva, sigue estas instrucciones:

1. **Revisar la Rúbrica:** Familiarízate con la estructura de la rúbrica antes de comenzar la evaluación.
2. **Responder en Secuencia:** Es mejor responder comenzando desde la pregunta 1 y continuar en orden. Sin embargo, si una pregunta en particular te inspira, siéntete libre de comenzar por ahí y luego aborda las preguntas restantes.
3. **Interpretar Cada Pregunta Cuidadosamente:** Tómate el tiempo para entender completamente la intención y el significado de cada pregunta antes de responder la evaluación.
4. **Entender los Niveles:** En cada pregunta, selecciona el nivel en el cual tu aplicas la estrategia de aprendizaje indicada, considerando el siguiente significado general de los niveles:
  • Nivel Muy Bajo: Prácticamente no hay implementación de la estrategia de aprendizaje.
  • Nivel Bajo: Uso ocasional y superficial de la estrategia de aprendizaje.
  • Nivel Medio: Ejecución regular de la estrategia de aprendizaje, pero sin profundidad.
  • Nivel Medio-Alto: Implementación constante y pertinente de la estrategia de aprendizaje.
  • Nivel Muy Alto: Aplicación continua, profunda y altamente relevante de la estrategia de aprendizaje indicada.
5. **Comenzar desde el Nivel Más Bajo:** Comienza tu evaluación en el nivel más bajo y avanza hacia arriba, de manera gradual. Marca con una “X" el nivel que refleje con precisión el grado en el cual tu aplicas cada estrategia de aprendizaje. Responde con honestidad; si solo se cumple parcialmente con los criterios de un nivel, considera el siguiente nivel más bajo como el nivel a elegir.
6. **Describir Logros y Aspectos para Mejorar:** Debajo de cada pregunta anota logros específicos y aspectos que se podrían mejorar en la aplicación de cada estrategia de aprendizaje.
  ¡Buena suerte con la evaluación de tus estrategias de aprendizaje!

## Appendix G Escala de Relevancia de la Educación en Línea

### Presentación

Esta escala ha sido diseñada para evaluar la pertinencia y relevancia de los entornos de aprendizaje en línea en diferentes tipos de instituciones educativas, incluyendo universidades. La educación en línea se ha convertido en un complemento esencial de la educación presencial, especialmente en contextos donde la flexibilidad y accesibilidad son prioritarias. Esta herramienta, diseñada desde la socioformación, permite medir aspectos críticos como la satisfacción de los estudiantes, la utilidad de las actividades en línea, los procesos adaptativos, y el grado de interacción y organización dentro de las aulas virtuales. Su aplicación es fundamental para entender cómo estos factores influyen en la experiencia de aprendizaje de los estudiantes y ofrecer indicaciones claras para mejorar la implementación y administración de plataformas virtuales de formación.

### Propósitos de la Escala

1. **Medir la satisfacción y utilidad:** Evaluar cómo los estudiantes y docentes perciben la pertinencia y relevancia de su experiencia en línea.
2. **Identificar aspectos de mejora en el aula virtual:** Analizar áreas específicas como la amigabilidad del aula, su organización, y la interacción que se promueve, para proponer mejoras efectivas.
3. **Evaluar la adaptabilidad y atracción del aula virtual:** Determinar la capacidad de los entornos virtuales para adaptarse a las necesidades individuales de los estudiantes y motivar su participación continua.

#### Dirigida a:

• **Docentes:** Para que puedan comprender mejor las dinámicas de sus aulas virtuales y realizar ajustes pedagógicos.
• **Autoridades educativas:** Para supervisar y regular la calidad de las ofertas educativas en línea.
• **Estudiantes:** Para que sean conscientes de sus propias experiencias de aprendizaje en línea y puedan proporcionar retroalimentación constructiva.

#### Instrucciones para el Uso de la Escala

1. **Revisar la escala completa:** Familiarízate con cada uno de los indicadores antes de comenzar la evaluación.
2. **Evaluar de forma secuencial o selectiva:** Puedes proceder evaluando desde el primer indicador o seleccionar un área específica que desees evaluar primero.
3. **Reflexionar sobre cada indicador:** Tómate el tiempo necesario para considerar cómo cada aspecto se aplica en tu contexto antes de realizar la valoración.
4. **Seleccionar el nivel apropiado:** Marca el nivel que mejor refleje la situación actual del aula virtual en cada uno de los indicadores propuestos, desde “Nivel Muy Bajo” hasta “Nivel Muy Alto".

#### Toma en cuenta el siguiente significado general de los niveles:

• **Nivel Muy Bajo:** No existe ninguna implementación pertinente del componente evaluado del aula virtual.
• **Nivel Bajo:** Implementación ocasional y limitada, sin contribuciones significativas al aprendizaje.
• **Nivel Medio:** Ejecución regular, cumpliendo con los requisitos básicos pero sin profundizar en la mejora del aprendizaje.
• **Nivel Medio Alto:** Implementación consistente y adecuada que apoya la pertinencia del proceso de aprendizaje.
• **Nivel Muy Alto:** Implementación excepcional y completa que maximiza el aprendizaje y la satisfacción.
5 **Describir logros y sugerencias:** Al final, describe los logros y los aspectos que deberían mejorarse en la plataforma en línea o en el aula virtual para aumentar su pertinencia.

Con la aplicación adecuada de esta escala, se pueden obtener una retroalimentación valiosa que permita enriquecer la experiencia educativa en línea, haciéndola más motivante y satisfactoria para todos los involucrados.

## Appendix H Online Education Relevance Scale

### Introduction

This scale is designed to assess the relevance and pertinence of online learning environments across various educational institutions, including universities. Online education has become an essential complement to face-to-face learning, particularly in contexts where flexibility and accessibility are paramount. Developed from a socioformation perspective, this tool measures critical aspects such as student satisfaction, the utility of online activities, adaptive processes, and the level of interaction and organization within virtual classrooms. Its application is crucial for understanding how these factors impact student learning experiences and for providing clear guidelines to enhance the implementation and management of virtual training platforms.

### Purposes of the Scale

1. **Measure satisfaction and utility:** Evaluate how students and teachers perceive the relevance and pertinence of their online experience.
2. **Identify areas for improvement in the virtual classroom:** Analyze specific areas such as classroom user-friendliness, organization, and the promoted interaction to propose effective enhancements.
3. **Assess virtual classroom adaptability and appeal:** Determine the virtual environments' ability to meet individual student needs and encourage ongoing participation.

#### Target Audience:

• **Educators:** To better understand the dynamics of their virtual classrooms and make pedagogical adjustments.
• **Educational authorities:** To monitor and regulate the quality of online educational offerings.
• **Students:** To be aware of their own online learning experiences and provide constructive feedback.

#### Instructions for Using the Scale

1. **Review the complete scale:** Familiarize yourself with each indicator before beginning the assessment.
2. **Evaluate sequentially or selectively:** You may proceed by evaluating from the first indicator or select a specific area you wish to evaluate first.
3. **Reflect on each indicator:** Take the necessary time to consider how each aspect applies in your context before making an assessment.
4. **Select the appropriate level:** Mark the level that best reflects the current situation of the virtual classroom for each proposed indicator, from “Very Low Level” to “Very High Level."
  • **Very Low Level:** No relevant implementation of the evaluated virtual classroom component exists.
  • **Low Level:** Occasional and limited implementation, with no significant contributions to learning.
  • **Medium Level:** Regular execution, meeting basic requirements but not enhancing learning.
  • **High Medium Level:** Consistent and appropriate implementation that supports the relevance of the learning process.
  • **Very High Level:** Exceptional and comprehensive implementation that maximizes learning and satisfaction.
5. **Describe achievements and suggestions:** At the end, describe the achievements and the aspects that should be improved in the online platform or virtual classroom to increase its relevance.

Proper application of this scale can provide valuable feedback that enriches the online educational experience, making it more motivating and satisfactory for all involved.
