# Supplementary material for: Development of transversal skills in higher education programs in conjunction with online learning: relationship between learning strategies, project-based pedagogical practices, e-learning platforms, and academic performance
Source: Heliyon. 2024 Dec 9;11(2):e41099. doi: 10.1016/j.heliyon.2024.e41099 (PMC11786650; doi:10.1016/j.heliyon.2024.e41099)
Supplement: Multimedia component 1 [file mmc1.pdf]

QUESTIONNAIRE ON SOCIODEMOGRAPHIC FACTORS, LEARNING RESOURCES AND ACADEMIC PERFORMANCE

Author: CIFE, 2020

www.cife.edu.mx

APA 7th Edition Reference:

CIFE (2020). Questionnaire On Sociodemographic Factors, Learning Resources, and Academic Performance. CIFE. https://www.cife.edu.mx/recursos

Instructions:

We invite you to complete a series of general sociodemographic questions to process the survey. All information is confidential.

Thank you.

1. Gender:

**Female**	**Male**
☐	☐

2. Indicate which age range you fall into:

**15-18**	**19-20**	**21-23**	**24-26**	**27-30**	**31-40**	**41-50**	**51-65**	**over 65 years**
☐	☐	☐	☐	☐	☐	☐	☐	☐

3. Indicate your age: ______ years
4. Marital Status:

**Single**	**Married**	**Domestic partnership**	**Divorced**	**Widowed**
☐	☐	☐	☐	☐

5. Do you have children?

0	1	2	3 or more
☐	☐	☐	☐

6. Level you are currently studying:

Undergraduate degree or bachelor's degree	Master's degree	Doctorate	Continuing education course
☐	☐	☐	☐

7. Semester you are currently in:

1	2	3	4	5	6	7	8	9	10	11 or higher
☐	☐	☐	☐	☐	☐	☐	☐	☐	☐	☐

8. Modality in which you are enrolled:

On-campus	Blended	Distance learning	Online
☐	☐	☐	☐

9. What was your approximate grade point average for the past semester? _____
10. What is your approximate grade point average for this semester? _____
11. In how many courses from the previous semester did you obtain a very high grade, above 9.0?

None	1 course	2 courses	3 courses	More than 3 courses
☐	☐	☐	☐	☐

12. In how many courses this semester do you have a very high grade, above 9.0?

None	1 course	2 courses	3 courses	More than 3 courses
☐	☐	☐	☐	☐

13. How many courses did you not pass last semester?

None	1	2	3	4	5	6	More than 6 courses or training projects
☐	☐	☐	☐	☐	☐	☐	☐

14. How many courses will you not pass this semester?

None	1	2	3	4	5	6	More than 6 courses or training projects
☐	☐	☐	☐	☐	☐	☐	☐

15. How many times have you withdrawn from studies and re-enrolled?

I have never withdrawn	Once	2-3 times	4-6 times	More than 6 times
☐	☐	☐	☐	☐

16. How many hours per week do you work in a company or business? (Work is considered any activity for which you receive economic income)

0 hours. I do not work	1-10 hours per week	11-20 hours per week	21-30 hours per week	31-40 hours per week	41-50 hours per week	51-60 hours per week	61-70 hours per week	More than 70 hours per week
☐	☐	☐	☐	☐	☐			

CUESTIONARIO DE FACTORES SOCIODEMOGRÁFICOS, RECURSOS PARA EL APRENDIZAJE Y DESEMPEÑO ACADÉMICO

(QUESTIONNAIRE ON SOCIODEMOGRAPHIC FACTORS, LEARNING RESOURCES, AND ACADEMIC PERFORMANCE)

Autor: CIFE, 2020

www.cife.edu.mx

APA 7th Edition Reference:

CIFE (2020). Questionnaire On Sociodemographic Factors, Learning Resources, and Academic Performance. CIFE. https://www.cife.edu.mx/recursos

Instrucciones:

Le invitamos a completar una serie de preguntas generales de tipo sociodemográfico para procesar la encuesta. Toda la información será confidencial.

Gracias

1. Género: | Mujer | Hombre | |-------|--------| | □ | □ |

2. Indica en qué rango está tu edad:

15-18	19-20	21-23	24-26	27-30	31-40	41-50	51-65	más de 65 años
□	□	□	□	□	□	□	□	□

3. Indica tu edad: _____ años

4. Estado civil:

Soltero	Casado	Unión libre	Divorciado	Viudo
□	□	□	□	□

5. ¿Tienes hijos? (Si su respuesta es No, la opción a seleccionar es "0")

0	1	2	3 o más
□	□	□	□

6. Nivel que cursas:

Carrera o licenciatura	Maestría	Doctorado	Curso de formación continua
☐	☐	☐	☐

7. Semestre que cursas:

1	2	3	4	5	6	7	8	9	10	11 o más
☐	☐	☐	☐	☐	☐	☐	☐	☐	☐	☐

8. Modalidad en la cual tienes matrícula:

Presencial	Semipresencial	A distancia	En línea
☐	☐	☐	☐

9. ¿Cuál fue tu promedio de calificaciones obtenido en el semestre pasado, de manera aproximada? _____

10. ¿Cuál es tu promedio aproximado de calificaciones este semestre? _____

11. ¿En cuántas asignaturas del semestre anterior obtuviste una calificación muy alta, superior a 9.0?

Ninguna	1 asignatura	2 asignaturas	3 asignaturas	Más de 3 asignaturas
☐	☐	☐	☐	☐

12. ¿En cuántas asignaturas del presente semestre tienes una calificación muy alta, superior a 9.0?

Ninguna	1 asignatura	2 asignaturas	3 asignaturas	Más de 3 asignaturas
☐	☐	☐	☐	☐

13. ¿Cuántas asignaturas no certificaste el semestre pasado?

Ninguna	1	2	3	4	5	6	Más de 6 asignaturas o proyectos formativos
☐	☐	☐	☐	☐	☐	☐	☐

14. ¿Cuántas asignaturas no vas a certificar este semestre?

Ninguna	1	2	3	4	5	6	Más de 6 asignaturas o proyectos formativos
☐	☐	☐	☐	☐	☐	☐	☐

15. ¿Cuántas veces te has retirado de los estudios y te has vuelto a inscribir?

Nunca me he retirado	1 vez	2-3 veces	4-6 veces	Más de 6 veces
☐	☐	☐	☐	☐

16. ¿Cuántas horas a la semana trabajas en alguna empresa o negocio? (si no trabaja, la respuesta es "0"). Se considera trabajo cualquier actividad por la cual se recibe algún ingreso económico.

0 horas. No trabajo	1-10 horas a la semana	11-20 horas a la semana	21-30 horas a la semana	31-40 horas a la semana	41-50 horas a la semana	51-60 horas a la semana	61-70 horas a la semana	Más de 70 horas a la semana
☐	☐	☐	☐	☐	☐	☐	☐	☐
